# Individual differences in cooperative and competitive play strategies

**DOI:** 10.1371/journal.pone.0293583

**Published:** 2023-11-09

**Authors:** Theresa C. Hauge, Daniel P. Ferris, Rachael D. Seidler

**Affiliations:** 1 Department of Applied Physiology & Kinesiology, College of Health and Human Performance, University of Florida, Gainesville, FL, United States of America; 2 J. Crayton Pruitt Family Department of Biomedical Engineering, Herbert Wertheim College of Engineering, University of Florida, Gainesville, FL, United States of America; Universitat Jaume I, SPAIN

## Abstract

**Introduction:**

Cooperation and competition are common in social interactions. It is not clear how individual differences in personality may predict performance strategies under these two contexts. We evaluated whether instructions to play cooperatively and competitively would differentially affect dyads playing a Pong video game. We hypothesized that instructions to play cooperatively would result in lower overall points scored and differences in paddle control kinematics relative to when participants were instructed to play competitively. We also predicted that higher scores in prosociality and Sportspersonship would be related to better performance during cooperative than competitive conditions.

**Methods:**

Pairs of participants played a Pong video game under cooperative and competitive instructions. During competitive trials, participants were instructed to score more points against one another to win the game. During the cooperative trials, participants were instructed to work together to score as few points against one another as possible. After game play, each participant completed surveys so we could measure their trait prosociality and Sportspersonship.

**Results:**

Condition was a significant predictor of where along the paddle participants hit the ball, which controlled ball exit angles. Specifically, during cooperation participants concentrated ball contacts on the paddle towards the center to produce more consistent rebound angles. We found a significant correlation of Sex and the average points scored by participants during cooperative games, competitive games, and across all trials. Sex was also significantly correlated with paddle kinematics during cooperative games. The overall scores on the prosociality and Sportspersonship surveys were not significantly correlated with the performance outcomes in cooperative and competitive games. The dimension of prosociality assessing empathic concern was significantly correlated with performance outcomes during cooperative video game play.

**Discussion:**

No Sportspersonship survey score was able to predict cooperative or competitive game performance, suggesting that Sportspersonship personality assessments are not reliable predictors of cooperative or competitive behaviors translated to a virtual game setting. Survey items and dimensions probing broader empathic concern may be more effective predictors of cooperative and competitive performance during interactive video game play. Further testing is encouraged to assess the efficacy of prosocial personality traits as predictors of cooperative and competitive video game behavior.

## 1. Introduction

### 1.1 Cooperation and competition in dyads

In two-person, or dyadic, social interactions, individuals can behave in cooperative, competitive, or neutral ways. Cooperation includes actions, attitudes, and beliefs that aim to benefit another individual, sometimes to achieve a shared or collective goal (e.g., Deutsch 1949a, 1949b; Simpson & Willer, 2017; for a review, see Rand & Nowak, 2013). Competition, in contrast, describes actions, attitudes, and beliefs contrary to those of another individual, with intentions that do not serve a shared goal or outcome [1, 2]. Cooperation and competition have been studied by having individuals play games that combine personal incentive with decisions that can affect a larger outcome and/or more individuals. This includes the use of the prisoner’s dilemma, public goods game, economic games, and other social dilemma paradigms [3–5]. For example, during a prisoner’s dilemma game, two players are faced with the decision to either cooperate with or defect from their partners, leading to different levels of monetary payoff depending upon whether one or both individuals cooperate or defect. These paradigms pose hypothetical scenarios in which players choose an outcome that is either mutually beneficial to both players or that prioritizes their own individual success. However, because the problems and consequences are not real and in these hypothetical scenarios, this approach may underestimate an individual’s cooperative versus competitive nature and its influence in real-world social interactions.

### 1.2 Understanding cooperative action as prosocial behavior

Cooperative actions are categorized within the broader group of prosocial behaviors, which have been studied in personality and social psychology [3]. Indeed, cooperation and cooperative interpersonal scenarios are commonly used as outcome measures of prosocial behaviors, attitudes, and beliefs. A review by Van Lange and colleagues (2013) suggested that prosocial personality and orientation has a tangible impact on the cooperative actions of an individual, and that priming with prosocial language improves the likelihood of cooperative outcomes in social interactions. Furthermore, those individuals considered more prosocial by their peers are perceived as cooperative during non-verbal interactions [5].

Individual differences in prosocial tendencies have been assessed for their association with various interpersonal interactions. One relevant example is the intersection of prosocial behavior, aggression, and violence in video games. Playing prosocial video games was shown to reduce hurtful behavior in a secondary task and to increase helpful actions when compared to violent games [6]. There is a negative association between prosocial video games and aggression/aggression-related variables [7]. The social context of game play also has a significant impact on prosocial behaviors even in violent video games; playing violent video games cooperatively can increase cooperative behaviors in subsequent interpersonal dilemmas [7–14]. Greater prosocial knowledge increases prosocial behavior in difficult social interactions [15] and mediates the relationship between prosocial behaviors, emotional intelligence, and agreeableness.

Traditionally, prosocial or helpful tendencies are assessed by asking individuals to rate their agreement with a list of statements that load onto different dimensions of helpful and other-oriented behaviors. For instance, Penner and colleagues constructed a prosociality personality battery by incorporating items from other assessments of personality (Penner, L. A., Fritzsche, B. A., Craiger, J. P., & Freifeld, T. S., 1995; in [16]). Penner and colleagues maintained that prosocial personality could not be described along one dimension, and that understanding individual differences in personality required a compilation of more heterogeneous items from scales assessing altruism, morality, empathy, and other facets of the personality.

### 1.3 Sportspersonship–prosocial personality in a sporting context

In recent years, psychologists have extended the assessment of prosociality to the sports context, asking participants to rate their alignment with statements reflecting different dimensions of Sportspersonship. For example, the Compliant and Principled Sportspersonship Scale (CAPSS) asks the individual to answer questions within a sporting context about beliefs and situations that can be generalized to many different sports, such as obeying officials, assisting injured opponents, or accepting criticism from a coach [17]. Prosocial behaviors are coded as cooperative–that is, they are helpful and beneficial to more than one actor [18–23]. Antisocial behaviors are frequently identified as those which are injurious toward the other actor; in some instances, competitiveness is linked to antisocial sporting behaviors [24]. These scales are combined with common social dilemma or behavior studies (e.g. using economic games or prisoner dilemma games) to assess the relationship between these dimensions and sport performance, identity, and motivation [25–27]. There is a positive relationship between prosocial behavior scores self-reported by athletes and measures of morality in sports [21–23, 28]. Prosocial behaviors also positively influence those who receive it; greater effort, performance, enjoyment, and commitment to the sport is related to prosocial behaviors on the field [29]. Sportspersonship scales have also been used to evaluate how prosocial behaviors transfer to team cohesion [30–33].

### 1.4 Individual differences in personality & motor behavior

Individual differences in prosociality and Sportspersonship have not been mapped to more discrete movements in sport that contribute to competitive or cooperative behavior. It is important to identify and quantify these actions because they may provide a more complete understanding of prosocial behaviors in live game play scenarios than through hypothetical scenarios such as the prisoner’s dilemma. Integrating the use of these batteries into motor control studies may provide a simple method of assessment for individual differences that can influence performance in cooperative or competitive situations. Understanding how individual differences in stable traits relate to performance under cooperative and competitive conditions can be used to optimize human performance via targeted selection and training for specific tasks.

Previous work in motor rehabilitation and neuroengineering leveraged Pong in cooperative and competitive games using personality as a covariate for performance in arm rehabilitation protocols. In these studies, personality predicted personal game preference between cooperative and competitive games in one-player and two-player modes for both impaired and unimpaired players [34, 35]. Personal preference for gameplay influenced overall rehabilitation motivation and engagement, which may improve rehabilitation outcomes. Additionally, interpersonal game play was generally preferred over computer-controlled opponents and increased motivation and engagement during game play [34, 36].

There are some key differences between these studies and the project presented here. While personality traits were able to predict personal preference for game type in most participants, the outcomes of interest did not use personality scores to compare game behaviors in competitive and cooperative games. The only metric used to evaluate performance across conditions was total point differences within and between subjects. One study also paired the impaired subject with a friend/relative or an occupational therapist in the clinical rehabilitation program; the presence of a previous relationship between players could have also contributed to game preferences and performance [14, 34].

### 1.5 Interpreting the kinematics of social interaction

Cooperation and competition behaviors have also been studied using motor tasks [37–41]. In reaching and grasping joint action tasks, participants exhibit different kinematic patterns depending on whether they are performing cooperatively or competitively [37, 39, 42]. Humans can distinguish between these social intentions based on the early kinematic data of a reaching task, even when provided with limited visual information [37, 43]. In one study, researchers examined the ability of participants to correctly classify the social intention in videos of a person performing solitary reach-to-grasp movements or social reach-to-grasp actions with a human partner. Participants could decode social intention from personal intention using the kinematic movement deviations present during a reach-to-grasp task [40]—total movement duration of a social intention condition was longer than for the personal intention condition. In addition, peak velocity was also slower than for personal intention reach-to-grasp actions. These individual differences in classification ability were predicted using a social skills questionnaire which measures a participant’s sensitivity to social cues [40]. This evidence suggests that there is significant interplay between dyadic motor tasks and individual differences in social cognition that may be identified using a combination of kinematic and psychological variables.

There is also evidence that kinematic behaviors are imitated during joint action tasks, even if the action imitation does not increase the likelihood of success [44–46]. Kinematic patterns can be influenced by task-irrelevant observation in joint task conditions. This imitation is more likely to occur during action observation scenarios [44], and in some cases the participant reaction time and movement distances can also strongly correlate with each other [46]. This unconscious imitation of observed movements translated to other competitive two-player games [46] and during interactive cooperative movement tasks with a virtual partner [45].

To summarize, the existing work on cooperative and competitive motor behaviors has largely been conducted using reach to grasp movements [39, 40], or actions that are meant to either be synchronized with or to mirror those of another participant [37]. This limits the generalizability of these findings to other movement types. A notable exception is work examining individual differences in motivation incentives (i.e., achievement versus affiliation bias) and how they relate to live sport / game performance. Sorrentino and Sheppard (1978) demonstrated that swimmers with higher affiliation trait scores performed better in a team relay context while those with higher achievement scores excelled in individual competition [47]. Another study used the Multi-Motive-Grid [48] to assess power and affiliation motive strengths and similarly found that those with higher affiliation motives performed better in a team darts competition than individually [49].

We investigated how individual differences in self-reported prosocial and Sportspersonship tendencies related to live, dyadic, video game play using a variant of the classic Pong game. Cooperative and competitive mindsets were induced with instructions and measured in terms of movement kinematics and points scored.

We expected that individual differences in performance would be predicted by prosocial and Sportspersonship traits. Specifically, we expected to see individuals with higher scores in prosociality and Sportspersonship to be better performers in the cooperative versus the competitive condition. We hypothesized that better performance in cooperative trials would be marked by longer bouts of consecutive Pong paddle hits and fewer points scored against the other player. We also hypothesized that location of ball contact along a player’s paddle (which relates to ball exit angle) would reflect strategy and planning, resulting in more predictable game play under the cooperative than competitive condition.

## 2. Methods and materials

### 2.1 Participants

A total of 20 pairs of young adults (n = 40, F = 22, age = 20.55 ± 2.67 years) were recruited from the University of Florida and greater Gainesville community. Participants were right-handed, and screened out via self-report for neurological deficits, upper limb injury, or history of traumatic brain injury. Participants were asked to report their relationship with one another to ensure no prior relationship or familiarity with the partner would impact performance during the session.

### 2.2 Experimental protocol

We paired participants for the experimental session using gender matching. Research of gender differences in cooperative and competitive play suggest that men experience stronger facial muscle physiological activation and overall positive emotional responses during competitive play vs cooperative play [50]. There is no such finding for women during either cooperative or competitive play [50].

**Fig 1:**
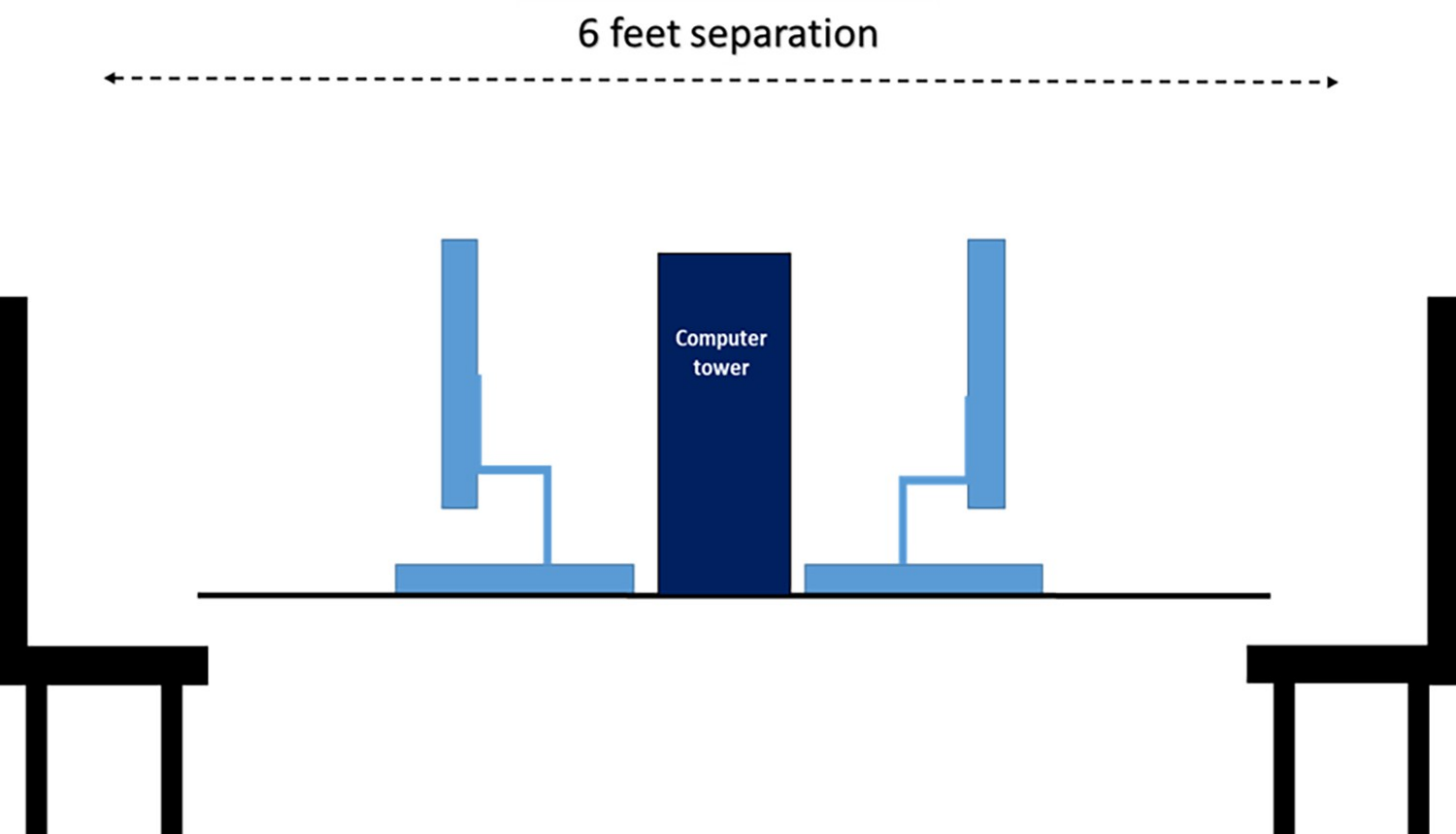
Illustration of experimental setup for game play between two persons in a laboratory setting. Participants were able to see each other over the computer monitors and central tower.

Participants first completed the consent form for the study, approved by the University of Florida Institutional Review Board (approval 202002080). After giving informed consent and completing an anonymous demographics form, participants were seated across from each other at two different computer monitors placed 6 feet apart on a table, separated by the desktop tower (see Fig 1). A member of the research team read a scripted explanation of the game to be played during the experiment–in this case, a version of the classic 1972 game Pong. After explaining the game to participants, a video of the game was presented on the computer monitor for reference.

Next, the researcher read aloud a written, scripted prompt for cooperative or competitive game play, delivered to pairs of participants in a counterbalanced order. Each participant pair performed both competitive and cooperative games with one another in the single session. A written copy of the prompts was available for participants if desired (see S1 Appendix). The prompts were used to: i) create a cooperative or competitive atmosphere with the wording and task instructions for that set of trials, as well as ii) discourage any voluntary vocalizations that may confound performance. Limiting vocalizations during game play was selected by researchers to compare these findings to future work; players were allowed to communicate between each game. Each session consisted of a familiarization game (three minutes in duration) followed by six, three-minute trials. Participants were given 30–60 seconds of break between each trial.

When all trials were completed for both sets of conditions, participants completed surveys in the presence of the researcher via pen and paper. They were administered i) the modified Penner Prosocial Personality Battery (PSB-27), a questionnaire that asks about qualities and behaviors considered prosocial, along two factors (other-oriented empathy and helpfulness); ii) the Multidimensional Sportspersonship Orientation Scale (MSOS-25); and iii) the Compliant and Principled Sportspersonship Scale (CAPSS-24). Although the MSOS-25 was included because of its frequent use in sport psychology studies [51–53], the CAPSS-24 was designed in 2015 to account for prior concerns about the internal validity of one dimension of the MSOS-25 [54]. Upon conclusion of the session, researchers compensated both participants equally for their time at a flat rate of $10/hour for the duration of the experiment.

### 2.3 Pong computer game

Traditionally, Pong is played with two players, each of whom control their own paddle on either side of the screen. In this version of the game, each participant controlled the upward and downward movement of their paddle, with the goal of contacting the ball that moves across the screen. Performance in cooperative trials was assessed by the number of cumulative points acquired between players–a lower number means that they were able to play more cooperatively. Performance in the competitive condition was assessed by the end score of the game–the player with the most points won the trial, and the player with the most points overall was declared the winner of that condition.

#### 2.3.1 Mechanics

Each player controlled their own paddle for the duration of the game. Participants moved the paddle up and down the screen using two adjacent keys on the keyboard. The upper key moved the paddle toward the top of the screen, and the lower key moved it to the bottom of the screen. Participants used the index and middle fingers of their dominant hand for keypresses.

#### 2.3.2 Ball movement

The ball moved continuously around the screen, ricocheting off the four walls and participant paddles. Participants controlled the direction of ball travel based on where it hit the paddle. The ball accelerated as it was hit back and forth between the players. Once a point was scored, the ball speed reset [55] with no pause or change in the trajectory of the ball.

**Fig 2:**
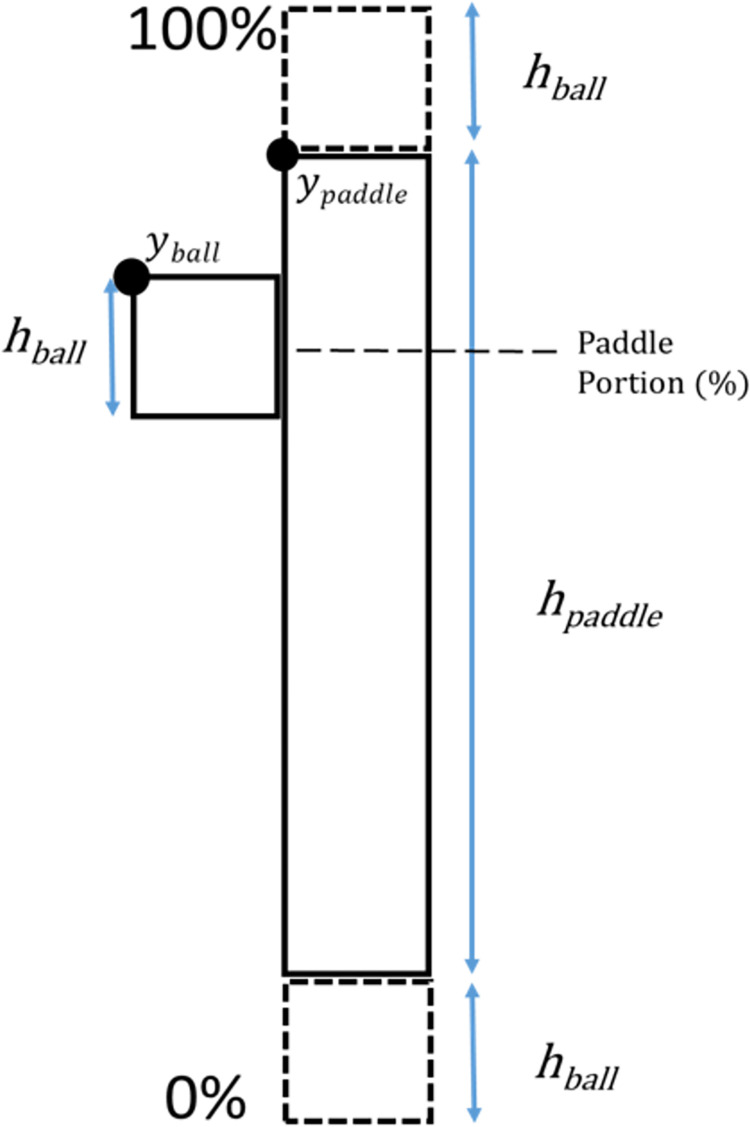
Schematic of ball-paddle contact values, and subsequent conversion to paddle portion (%). The location (in pixels) of ball contact with the paddle was converted to a percentage or portion of the paddle that was used later in calculating the ball exit angle following contact.

**Fig 3:**
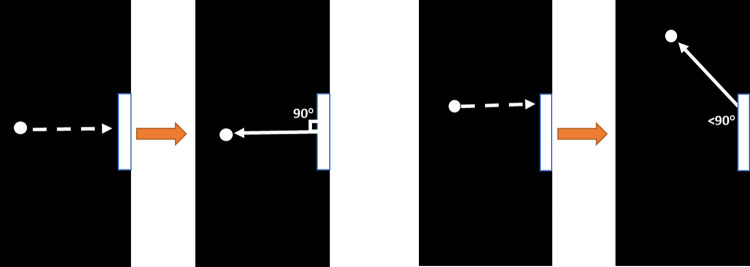
Illustrations to explain the result of the ball rebounding off different points of the paddle. In 3A, the ball approaches the center of the paddle and rebounds at a 90° angle relative to the paddle. Fig 3B shows the ball hitting a portion of the paddle further from the center and rebounding at an angle < 90°.

The ball exit angle (Eq 2) from the paddle was calculated in part using the paddle impact location (PIL, Eq 1A) between the ball and the paddle, measured as the location on the paddle in pixels. The PIL was converted to Paddle Portion (PP, Eq 1B) so that the PIL of the ball could be reinterpreted as ball impact along the paddle as a percentage, from the bottom of the paddle (0%) to the top of the paddle (100%) (see Figs 2 and 3).

$$Paddle\;Impact\;Location\;(PIL)/y_{impact}={(y}_{ball}+h_{ball})-y_{paddle}$$ Eq 1A

Where *y*_*ball*_ = y-coordinate of the ball, *y*_*paddle*_ = y-coordinate of the paddle, *h*_*ball*_ = ball height (15 pixels), *h*_*paddle*_ = paddle height (150 pixels), and PIL = paddle impact location

$$Paddle\;Portion\;(PP,\%)=\left(\frac{PIL}{h_{paddle}+{2h}_{ball}}\right)\times 100\%$$ Eq 1B

PIL was divided by *h*_*paddle*_ = paddle height (150 pixels), and *h*_*ball*_ = ball height (15 pixels) at contact. This value was subsequently converted to a percentage representing contact with the bottom (0%), top (100%), or other areas of the paddle.

After converting the global location of impact (PIL) to its local, equivalent portion of the paddle (PP), this value was included in the calculation of the ball exit angle from the paddle. The PP value acts as a multiplier of the total range of ball exit angles during a game, and helps determine the direction (upwards or downwards) of the ball exit angle:

$$Ball\;Angle\;()=({range}_{angle}\times PP\%-(\frac{{range}_{angle}}{2})+int[-5{}^{\circ},5{}^{\circ}]),$$ Eq 2

Where *range*_*angle*_ = range of ball angle on bounce, *PP%* = paddle portion, and *int[-5°*,*5°]* = randomization value added to ball angle, between -5° and 5°. It should be noted that the origin–that is, 0 degrees–is modified so that all upward deflections yield angles less than 90 degrees, and all downward deflections from the paddle yield angles greater than 90 degrees. This is applied to the ball bounce angle regardless of which paddle it has contacted. The ball exit angle from the paddle was modified by adding a randomized integer value designed to reduce unrealistic predictability of the ball path. Specifically, this randomization eliminated the likelihood of participants using an optimal alignment of their paddles to send the ball between them at exactly 90° without need for intervention or active engagement in the game.

### 2.4 Surveys and questionnaires

#### 2.4.1 Compliant and Principled Sportspersonship Scale (CAPSS-24)

The CAPSS-24 is a 24-item survey that asks participants to rate statements that code to five factors of Sportspersonship: i) compliance with officials; ii) compliance towards rules; iii) not legitimizing injurious acts; iv) respect for opponent; and v) game perspective. This survey specifies answers within the sport domain, which can provide insight into state behaviors during a sporting context.

The Compliant and Principled Sportspersonship Scale (CAPSS) was developed in 2014 to account for issues identified with the MSOS [17, 54]. The CAPSS includes factors and items from moral behavior literature linking morality to Sportspersonship [18, 56, 57], that were previously absent in the MSOS. The creators of the CAPSS administered their scale to participants playing competitive and accumulative rounds of the prisoner’s dilemma game. They found that the CAPSS predicted cooperation percentages in participants when their actions lead to future consequences [21]. Comparatively, the CAPSS is not used or cited as frequently as other Sportspersonship surveys (e.g. PABSS, MSOS, etc.), but was a promising addition to explore prosocial tendencies via Sportspersonship, with more attention paid to issues of morality and other-oriented reasoning [58–61].

#### 2.4.2 Multiple Sportspersonship Orientation Scale (MSOS-25)

The Multidimensional Sportspersonship Orientation Scale (MSOS) was developed by Vallerand and colleagues (1997) and is still used in many sports psychology studies [51–53, 62]. However, the MSOS is an imperfect model of Sportspersonship. One of the five subscales has low internal validity, and another subscale is critiqued for combining two mutually exclusive dimensions into one [63]. This model also lacks items assessing the person’s morality, which can influence a person’s behavior in sport, whether as a proactive or inhibitive mechanism [20, 57].

The MSOS-25 is a 25-item survey that asks participants to rate statements that are related to five different types of Sportspersonship–i) concern and respect for the opponent; ii) respect for social conventions; iii) respect for the rules and the officials; iv) respect for one’s full commitment toward sport participation; and v) negative approach toward the practice of sport [64].

#### 2.4.3 Penner Prosocial Battery, revised (PSB-27)

The PSB is a 56-item questionnaire that asks individuals to rate various statements in relation to their own beliefs and behaviors and provides insight into the dispositional tendencies of an individual towards prosociality. In generating the items of the PSB, scales correlating with prosocial attitude, thinking, and actions were considered only if the theoretical model of the personality scale provided an explanation connecting the characteristic to prosocial tendencies [65]. The PSB is administered in other social psychology experiments such as the prisoner’s dilemma or economic goods games, and the effect of violence or aggression on subsequent prosocial decisions made in video games has also been measured using this survey [13].

Subscales include social responsibility, empathic concern, perspective taking, personal distress, other-oriented moral reasoning, mutual concerns moral reasoning, and self-reported altruism. These subscales load into two larger factors, Other-Oriented Empathy, and Helpfulness. Other-Oriented Empathy is a factor that relates most closely with the internal cognition and affectation of empathy and the relationship to prosocial thoughts and feelings. Helpfulness items relate most closely to the prosocial behaviors and actions of individuals. These are all elements of social interactions that have clear overlap with other prosocial behaviors such as cooperation during a two-person motor task. We administered a revised version of the PSB-30, consisting of 27 items across all subscales except personal distress.

### 2.5 Pong performance measures

For both cooperation and competition conditions we measured the points earned by each participant across trials. In addition to measuring points scored to assess performance, we also measured the ball contact position along the length of the paddle, referred to as **paddle portion (PP)** (see Eqs 1A, 1B). Mean ball contact position (PP_mean_) and standard deviation (PP_SD_) during trials were examined for evidence of differential strategy engagement between cooperation and competition. To study the absolute distance of ball contacts from the center of the paddle (PP_diffABS_), we performed the following calculation: PP_diffABS_ = | PP_*i*_−PP_center_ |, where *i* is the paddle portion of each paddle contact that occurs during a round of gameplay and PP_center_ = 50%, aka the center of the paddle. The center of the paddle translated to a perpendicular (90 degree) exit angle of the ball off the paddle.

### 2.6 Statistical analysis

To control for random effects of Participant on points scored across the data collection, we selected a linear mixed model for analysis (run in R (version 4.0.2) using the lme4() package [66]):

$$y=X_{n}\beta_{n}+Zu+\varepsilon$$

In this model, *y* is the outcome variable, *X* and *Z* are design matrices of observations relating *β* and *u* to the outcome variable y; *β* represents unknown fixed effects of predictor variables; *u* is a matrix/vector of unknown random effects; and ε represents an unknown vector of random errors.

In a linear mixed effect model, assumptions of normality apply to the model residuals. The fixed effects of condition, trial, sex, and all three survey scores were added to the model, as well as the Condition*Survey interactions for each survey. Significance was calculated using the lmerTest package [67], which applies Satterthwaite’s method to estimate degrees of freedom and generate p-values for mixed models [68]. The random effect of Subject was included in each model.

To assess changes in game strategy, we compared paddle portion between cooperative and competitive conditions. We considered the average (PP_mean_) and standard deviation (PP_SD_) of collision percentage from all ball contacts, as well as the absolute distance (PP_diffABS_) of ball collisions from the paddle center (center PP = 50%). The same linear mixed model analysis for points scored was applied to all collision percentage analyses. The final model specifications were as follows:

$$y=X_{1}\beta_{1}+X_{2}\beta_{2}+\cdots X_{n}\beta_{n}+Zu+\varepsilon$$

Where *y* = Performance Outcome (Points Scored, PP_mean_, PP_SD_, PP_diffABS_); *X*_*n*_ is the design matrix of observations for *β*; *β*_*n*_ is a matrix of unknown fixed effects for the predictor variables Condition, Trial, Sex, Surveys (CAPSS, MSOS, PSB), and the Condition*Survey interactions; and *Z* is the design matrix for *u*, a vector of unknown random effects of Subject. The variables Condition (competition/cooperation), and Sex (male/female) were dummy coded for ease of interpretation as factors in each model.

To correct for non-normal model residuals, survey scores were rank ordered and replaced the original values in the model [66]. Because the model residuals for all outcome variables were not normally distributed after rank-transforming predictors (see Supporting Information S3), we used nonparametric Spearman rank correlations [69] using the cor.test() function in the R package ‘stats’ [70] to assess the relationship between the outcome variables averaged across all condition trials and predictor variables for all trials performed by the participants. The p-values were adjusted for multiple comparisons using the Benjamini-Hochberg correction using p.adjust() (‘stats’ package, R) to control the false discovery rate (FDR) [71]. The predictors Sex and Condition were coded for analyses so that [women = 1, men = 0] and [cooperation = 1, competition = 0]. A non-parametric Friedman test was used to assess the effect of trial on each outcome variable in cooperation and competition [72].

## 3. Results

### 3.1 Subject demographics and survey results

Subject demographics and descriptive statistics for survey scores are presented in Tables 1 and 2. A higher score on a survey reflects either greater prosocial tendencies (PSB) or higher Sportspersonship (MSOS, CAPSS). Welch’s two-sample unpaired t-tests [70] showed a significant difference in means between women and men for age and all survey scores (p < 0.01).

**Table 1:** Subject demographics and group mean ± standard deviation of survey scores. Includes maximum earnable points for each survey, survey scores grouped by sex, and results of unpaired two-sample t-tests. PSB-27 = Penner Prosocial Battery, MSOS-25 = Multidimensional Sportspersonship Orientation Scale, CAPSS-24 = Compliant and Principled Sportspersonship Scale. *** = p < 0.001.

**Total Sample Size**		**# Women**	**# Men**	**p-value (t-test)**
N = 40		N_women_ = 22	N_men_ = 18	
Age ± SD (years)		Age ± SD (years)	Age ± SD (years)	< 2.2e-16 ***
20.6 ± 2.7		20.9 ± 3.3	20.1 ± 1.4	
**Survey**	**Maximum Possible Score**	**Mean ± SD**	**Mean ± SD**	**p-value (t-test)**
PSB-27	135	99.8 ± 9.29	95.1 ± 8.3	< 2.2e-16 ***
MSOS-25	125	82.9 ± 13.97	87.6 ± 12.9	< 2.2e-16 ***
CAPSS-24	120	102.8 ± 7.7	96.7 ± 10.1	< 2.2e-16 ***

**Table 2:** Descriptive statistics of survey scores; mean, standard deviation, median (25^th^/75^th^ percentile), range, minima (min) / maxima (max) of survey results, skewness (Skew), kurtosis, and standard error (SE). PSB-27 = Penner Prosocial Battery, MSOS-25 = Multidimensional Sportspersonship Orientation Scale, CAPSS-24 = Compliant and Principled Sportspersonship Scale.

Survey	Mean	SD	Median (25, 75^th^ %ile)	Min	Max	Range	Skew	Kurtosis	(SE)
**PSB-27**	97.7	9.17	95.5 (91, 105.5)	81	114	33	0.05	-1.066	0.59
**MSOS-25**	85	13.7	89.5 (74.5, 94.5)	48	108	60	-0.84	-0.026	0.88
**CAPSS-24**	100.1	9.38	102 (94, 107.3)	77	120	43	-0.29	-0.53	0.61

### 3.2 Spearman’s rank correlations

Here we report the results of the Spearman rank correlations for the overall survey score and the scores of the dimensions within the survey. The Spearman rank correlations that originally were significant predictors before Benjamini-Hochberg correction are reported in Tables 3–11. All tables of correlations can be found in Supporting Information S1 File.

**Table 3:** All spearman correlations of outcome variables and total CAPSS survey score. All trials = averaged across all trials for participant, cooperation = average value of cooperation trial performance; competition = average value of competition trial performance. Bolded p-values indicate significance.

Outcome	Predictor	Spearman’s rho (ρ)	p-value	Adjusted p-value (B-H)
Average Points, cooperation	CAPSS	.29	0.067	0.543
PP_SD_, competition	CAPSS	-.28	0.081	0.543
Average Points, all trials	CAPSS	.22	0.172	0.543
PP_diffABS_, competition	CAPSS	-.19	0.251	0.543
PP_mean_, competition	CAPSS	.18	0.2697	0.543
PP_SD_, all trials	CAPSS	-.17	0.3071	0.543
PP_diffABS_, cooperation	CAPSS	.16	0.3149	0.562
Average Points, competition	CAPSS	.15	0.3471	0.622
PP_mean_, all trials	CAPSS	.11	0.4828	0.622
PP_diffABS_, all trials	CAPSS	-.05	0.7496	0.623
PP_SD_, cooperation	CAPSS	.03	0.8629	0.623
PP_mean_, cooperation	CAPSS	3.6E-03	0.9825	0.640

**Table 4:** Results of all outcome variables and the CAPSS dimension game perspective. All trials = averaged across all trials for participant, cooperation = average value of cooperation trial performance; competition = average value of competition trial performance. Bolded p-values indicate significance.

Outcome	Predictor	Spearman’s rho (ρ)	p-value	Adjusted p-value (B-H)
Average Points, cooperation	CAPSS, game perspective	.36	0.023*	0.272
Average Points, all trials	CAPSS, game perspective	.31	0.052	0.311
Average Points, competition	CAPSS, game perspective	.23	0.146	0.565
PP_SD_, competition	CAPSS, game perspective	-.21	0.188	0.565
PP_diffABS_, cooperation	CAPSS, game perspective	.16	0.313	0.609
PP_SD_, all trials	CAPSS, game perspective	-.16	0.335	0.609
PP_mean_, all trials	CAPSS, game perspective	-.15	0.355	0.609
PP_mean_, cooperation	CAPSS, game perspective	-.12	0.457	0.685
PP_mean_, competition	CAPSS, game perspective	-.06	0.722	0.946
PP_diffABS_, competition	CAPSS, game perspective	-.04	0.788	0.946
PP_diffABS_, all trials	CAPSS, game perspective	.01	0.939	0.990
PP_SD_, cooperation	CAPSS, game perspective	-2.1E-03	0.990	0.990

**Table 5:** Results of all outcome variables and the CAPSS dimension legitimizing injurious acts. All trials = averaged across all trials for participant, cooperation = average value of cooperation trial performance; competition = average value of competition trial performance. Bolded p-values indicate significance.

Outcome	Predictor	Spearman’s rho (ρ)	p-value	Adjusted p-value (B-H)
PP_SD_, competition	CAPSS, not legitimizing injurious acts	-.38	**0.015**	0.075
PP_diffABS_, competition	CAPSS, not legitimizing injurious acts	-.37	0.018*	0.075
Average Points, cooperation	CAPSS, not legitimizing injurious acts	.37	0.019*	0.075
Average Points, both conditions	CAPSS, not legitimizing injurious acts	.33	0.036*	0.106
PP_mean_, all trials	CAPSS, not legitimizing injurious acts	.28	0.081	0.192
PP_mean_, competition	CAPSS, not legitimizing injurious acts	.26	0.100	0.192
Average Points, competition	CAPSS, not legitimizing injurious acts	.26	0.112	0.192
PP_SD_, all trials	CAPSS, not legitimizing injurious acts	-.24	0.140	0.209
PP_diffABS_, cooperation	CAPSS, not legitimizing injurious acts	.30	0.170	0.226
PP_mean_, cooperation	CAPSS, not legitimizing injurious acts	.20	0.224	0.268
PP_diffABS_, all trials	CAPSS, not legitimizing injurious acts	-.14	0.398	0.434
PP_SD_, cooperation	CAPSS, not legitimizing injurious acts	.02	0.923	0.923

**Table 6:** Results of all outcome variables and the MSOS survey score. All trials = averaged across all trials for participant, cooperation = average value of cooperation trial performance; competition = average value of competition trial performance. Bolded p-values indicate significance.

Outcome	Predictor	Spearman’s rho (ρ)	p-value	Adjusted p-value (B-H)
PP_diffABS_, cooperation	MSOS	-.27	0.098	0.543
Average Points, cooperation	MSOS	-.26	0.102	0.543
Average Points, all trials	MSOS	-.23	0.151	0.543
PP_SD_, cooperation	MSOS	-.22	0.184	0.543
Average Points, competition	MSOS	-.19	0.252	0.543
PP_SD_, all trials	MSOS	-.18	0.272	0.543
PP_SD_, competition	MSOS	-.16	0.328	0.562
PP_mean_, competition	MSOS	.13	0.419	0.622
PP_mean_, all trials	MSOS	-.11	0.518	0.622
PP_mean_, cooperation	MSOS	-.10	0.538	0.622
PP_diffABS_, competition	MSOS	-.09	0.571	0.622
PP_diffABS_, all trials	MSOS	-.08	0.640	0.640

**Table 7:** Results of all outcome variables and the MSOS dimension “respect for social convention”. All trials = averaged across all trials for participant, cooperation = average value of cooperation trial performance; competition = average value of competition trial performance. Bolded p-values indicate significance.

Outcome	Predictor	Spearman’s rho (ρ)	p-value	Adjusted p-value (B-H)
Average Points, cooperation	MSOS, respect for social conventions	-.41	0.008**	0.095
Average Points, all trials	MSOS, respect for social conventions	-.37	0.020*	0.118
Average Points, competition	MSOS, respect for social conventions	-.27	0.098	0.391
PP_diffABS_, cooperation	MSOS, respect for social conventions	-.22	0.181	0.483
PP_diffABS_, competition	MSOS, respect for social conventions	.21	0.201	0.483
PP_SD_, cooperation	MSOS, respect for social conventions	-.12	0.478	0.926
PP_mean_, competition	MSOS, respect for social conventions	-.07	0.672	0.926
PP_mean_, all trials	MSOS, respect for social conventions	-.07	0.678	0.926
PP_SD_, competition	MSOS, respect for social conventions	.05	0.739	0.926
PP_mean_, cooperation	MSOS, respect for social conventions	-.05	0.772	0.926
PP_SD_, all trials	MSOS, respect for social conventions	.012	0.943	0.989
PP_diffABS_, all trials	MSOS, respect for social conventions	2.4E-03	0.989	0.989

**Table 8:** Results of all outcome variables and the PSB survey score. All trials = averaged across all trials for participant, cooperation = average value of cooperation trial performance; competition = average value of competition trial performance. Bolded p-values indicate significance.

Outcome	Predictor	Spearman’s rho (ρ)	p-value	Adjusted p-value (B-H)
PP_diffABS_, competition	PSB	.18	0.256	0.908
PP_diffABS_, all trials	PSB	.17	0.303	0.908
Average Points, competition	PSB	.17	0.306	0.908
PP_SD_, cooperation	PSB	.14	0.402	0.908
Average Points, all trials	PSB	.13	0.428	0.908
PP_diffABS_, cooperation	PSB	.12	0.457	0.908
PP_SD_, all trials	PSB	.09	0.580	0.908
PP_mean_, cooperation	PSB	.08	0.646	0.908
Average Points, cooperation	PSB	.07	0.686	0.908
PP_SD_, competition	PSB	.05	0.774	0.908
PP_mean_, competition	PSB	.03	0.833	0.908
PP_mean_, both conditions	PSB	.02	0.913	0.913

**Table 9:** Results of all Spearman correlations between outcome variables and the PSB dimension “empathic concern”. All trials = averaged across all trials for participant, cooperation = average value of cooperation trial performance; competition = average value of competition trial performance. Bolded p-values indicate significance.

Outcome	Predictor	Spearman’s rho (ρ)	p-value	Adjusted p-value (B-H)
PP_diffABS_, cooperation	PSB, empathic concern	.48	0.002**	0.022*
PP_SD_, cooperation	PSB, empathic concern	.45	0.004**	0.022*
PP_diffABS_, all trials	PSB, empathic concern	.35	0.025*	0.097
Average Points, cooperation	PSB, empathic concern	.34	0.032*	0.097
Average Points, all trials	PSB, empathic concern	.31	0.051	0.122
PP_SD_, all trials	PSB, empathic concern	.28	0.081	0.162
Average Points, competition	PSB, empathic concern	.27	0.095	0.163
PP_mean_, competition	PSB, empathic concern	.18	0.273	0.410
PP_mean_, cooperation	PSB, empathic concern	-.12	0.459	0.565
PP_SD_, competition	PSB, empathic concern	.12	0.471	0.565
PP_diffABS_, competition	PSB, empathic concern	.05	0.757	0.825
PP_mean_, all trials	PSB, empathic concern	-.02	0.918	0.918

**Table 10:** Results of all outcome variables and sex. All trials = averaged across all trials for participant, cooperation = average value of cooperation trial performance; competition = average value of competition trial performance. Bolded p-values indicate significance.

Outcome	Predictor	Spearman’s rho (ρ)	p-value	Adjusted p-value (B-H)
Average Points, cooperation	Sex	.71	3.15E-07***	3.780E-06***
Average Points, all trials	Sex	.67	2.19E-06***	1.314E-05***
PP_diffABS_, cooperation	Sex	.59	6.69E-05***	2.676E-04***
Average Points, competition	Sex	.55	2.269E-04***	6.807E-04***
PP_SD_, cooperation	Sex	.43	5.500E-03**	0.013*
PP_diffABS_, all trials	Sex	.30	0.06364	0.127
PP_SD_, all trials	Sex	.20	0.2154	0.370
PP_mean_, competition	Sex	.19	0.2474	0.371
PP_mean_, cooperation	Sex	-.15	0.3479	0.464
PP_diffABS_, competition	Sex	-.11	0.4868	0.584
PP_SD_, competition	Sex	-.04	0.8103	0.873
PP_mean_, all trials	Sex	.03	0.8729	0.873

**Table 11:** Results of Friedman’s non-parametric test to assess the effect of trial on all outcome variables. All trials = averaged across all trials for participant, cooperation = average value of cooperation trial performance; competition = average value of competition trial performance. Bolded p-values indicate significance.

Outcome	Predictor	Chi-squared (χ^2^)	Degrees of freedom (df)	p-value
Points scored, cooperation	Trial	9.6524	5	0.08571
PP_mean_, cooperation	Trial	8.2571	5	0.1426
PP_SD_, cooperation	Trial	6.1143	5	0.2953
PP_diffABS_, cooperation	Trial	12.462	5	0.029*
Points scored, competition	Trial	6.8779	5	0.2299
PP_mean_, competition	Trial	4.5714	5	0.4704
PP_SD_, competition	Trial	10.086	5	0.07284
PP_diffABS_, competition	Trial	5.4571	5	0.3627

#### 3.2.1 Condition effect on game behaviors

Results of the spearman correlation between the outcome variables and condition (cooperation = 1) indicated that condition instructions were a significant predictor of the relative distance of paddle hits from paddle center (PP_diffABS_: *ρ* = -.58, p_adj_ = 6.24E-08) and participant paddle hit standard deviation (PP_SD_: *ρ* = -.43, p_adj_ = 1.45E-04) (see Table 12).

**Table 12:** Results of Spearman correlations of outcome variables and Condition as a predictor. PP_diffABS_ = paddle portion, absolute distance from paddle center; PP_SD_ = paddle portion, standard deviation of hits along paddle surface; PP_mean_ = paddle portion, mean paddle hit location. Bolded p-values indicate significance.

Outcome	Predictor	Spearman’s rho (ρ)	p-value	Adjusted p-value (B-H)
PP_diffABS_	Condition	-.58	1.56E-08***	6.24E-08***
PP_SD_	Condition	-.43	7.26E-05***	1.45E-04***
Points	Condition	-.19	0.085	0.113
PP_mean_	Condition	.17	0.124	0.124

#### 3.2.2 CAPSS survey and dimensions

The cumulative score on the CAPSS survey of Sportspersonship did not significantly predict participant outcome variables during cooperation or competition games, as well as average performance outcomes across both conditions (Table 3, p > 0.05). Prior to Benjamini-Hochberg correction, the “game perspective” dimension of the CAPSS was a significant predictor of average points scored during cooperation games (Table 4, ρ = .36, p = 0.023). There were also significant negative correlations between “not legitimizing injurious acts” (Table 5) and PP_SD_ (ρ = -.38, p = 0.015) and PP_diffABS_ (ρ = -.37, p = 0.018) in competitive games and positive correlations with average points scored in cooperative games (ρ = .37, p = 0.019) and average points scored across all games (ρ = .33, p = 0.036). These correlations did not remain significant after Benjamini-Hochberg correction (p_adj_ > 0.05).

#### 3.2.3 MSOS survey and dimensions

The cumulative score of the MSOS survey of Sportspersonship was not a significant predictor of any participant performance outcomes during cooperation, competition, or across all games played during the session (Table 6). There was a significant negative correlation between the dimension “respect for social convention” (Table 7) and average points scored in cooperative games (*ρ* = -.41, p = 0.008) and average points scored across all games (*ρ* = -.37, p = 0.020). However, these correlations did not remain significant following Benjamini-Hochberg correction for multiple comparisons.

#### 3.2.4 PSB survey and dimensions

The cumulative PSB survey score for prosociality was not a significant predictor of participant outcomes for cooperative games, competitive games, or for overall performance across the session (Table 8). The dimension “empathic concern” (Table 9) was significantly positively correlated with PP_diffABS_ (ρ = .48, p = 0.002) and PP_SD_ (ρ = .45, p = 0.004) during cooperative games, and remained significant after Benjamini-Hochberg correction for multiple comparisons (PP_diffABS_: p_adj_ = 0.022; PP_SD_: p_adj_ = 0.022). Prior to p-value correction, empathic concern was also significantly correlated with overall PP_diffABS_ for all games (*ρ* = .35, p = 0.025) and the average number of points scored during cooperative games (*ρ* = .34, p = 0.032); however, these correlations did not remain significant after Benjamini-Hochberg correction.

#### 3.2.5 Effect of Sex on performance outcomes

Sex was a significant predictor of outcome variables in cooperative games, competitive games, and also outcome variables assessing performance for all games.

#### 3.2.6 Effect of time on outcome variables

We used non-parametric Friedman’s testing to determine the effect of time on participant outcomes (Table 11). There was a significant effect of time for the absolute difference in paddle location from center during cooperation trials (*χ*^*2*^ *=* 12.462, p = 0.029).

## 4. Discussion

Personality research has revealed individual differences in cooperative and competitive tendencies; these have been shown to predict behavior in social contexts. Whether or how they relate to live game play has been less studied. Here, we used a two-person video game modified from the original Atari Pong game and manipulated social context to investigate individual differences in behavior. We included three personality surveys to examine the relationship between self-reported prosocial and Sportspersonship tendencies with performance.

### 4.1 Psychology surveys were not accurate predictors of cooperative or competitive motor performance

When assessing the primary performance outcomes for participants, no cumulative survey score was a significant predictor of cooperative or competitive performance outcomes during live game play following multiple comparison corrections. Each survey contained dimensions within the full survey that were initially significant predictors of game behaviors, but after Benjamini-Hochberg corrections only one dimension of the Prosocial Battery (PSB), “empathic concern”, was a significant predictor of motor behavior during cooperative game play. Penner et al. described the empathic concern dimension of the PSB as “the tendency to experience other-oriented feelings of sympathy and concern for unfortunate others” [65], with the original items selected from Davis’s Interpersonal Reactivity Index (IRI) [73, 74]. The results of this study could suggest that the motor behaviors adopted during cooperation are physical representation of one person’s greater empathic concern for the other player in that social context. This is an interesting finding because of the generalized nature of the PSB items. This scale includes a broad range of scenarios, but they generally do not relate to physical interactions or sport. For example, items include:

“With the pressure for grades and the widespread cheating in school nowadays, the individual who cheats occasionally is not really as much at fault.”

“When I see someone being taken advantage of, I feel kind of protective towards them.”

“My decisions are usually based on my concern for other people.”

“I have helped carry a stranger’s belongings.”

It is possible that this quality of the PSB makes it a more flexible assessment of personality, and therefore is a more effective survey for use in future studies incorporating the effect of individual differences on social motor behaviors. Future work can also include the Interpersonal Reactivity Index to compare the predictive power of the PSB dimension to the original multidimensional scale put forth by Davis [73, 74]

The Compliant and Principled Sportspersonship Scale (CAPSS) includes the addition of morality literature into the survey development, establishing a distinction between compliant and principled Sportspersonship behaviors unlike other Sportspersonship scales used in social psychology [20]. Compliant Sportspersonship describes behaviors that reflect a person’s adherence to expectations established by society during sport and game environments. Principled Sportspersonship, in contrast, describes behaviors that are dictated by the individual’s own moral values or principles. These environmental and personal morals may not always align, and understanding the composition of a player’s Sportspersonship can provide better insight into cooperative and competitive performance differences.

Within the context of this study, the CAPSS survey was not an effective predictor of cooperative or competitive behaviors during virtual game play. No dimensions of the CAPSS were significant predictors of participant motor behaviors during cooperative or competitive video game play. The uncorrected p-values of the CAPSS dimensions “game perspective” and “not legitimizing injurious acts” were less than 0.05, but they failed to reach the Benjamini-Hochberg corrected threshold p-value. Continued testing and application of the CAPSS to a variety of cooperative and competitive gaming conditions is encouraged.

No dimension of the MSOS was an adequate predictor of motor behavior outcomes during game performance. In their original study, Vallerand et al determined that specific subscales of the MSOS were more closely related to behavioral intentions than others, namely the social conventions subscale [75]. In this study, the dimension “social conventions” was only a significant predictor of average points scored during cooperation and across all games prior to multiple comparisons corrections.

It is surprising that the CAPSS and MSOS were not significant predictors of cooperative or competitive motor behaviors. These Sportspersonship surveys contain items that are broadly applicable to many sports and athletes, but do not report responses to specific motor behaviors that might occur in each sport, such as passing a ball to a teammate. To our knowledge, these surveys had never been applied to specific motor behaviors and strategies employed within a given sport. It is unknown, for instance, if there is a relationship between a player’s CAPSS or PSB score and pass frequency to other teammates during a game of soccer. It may be that these surveys are not sensitive enough to predict these in-game behaviors.

These results warrant further use of personality scales in other sporting and game studies to compare the effectiveness of Sportspersonship and prosociality to predict motor behaviors in sport.

### 4.2 The social context of video game play

We found that participants adjusted their strategies in response to cooperative or competitive play instructions, resulting in differential paddle kinematics between conditions. Participants varied the location of ball contacts on their respective paddles for more or less predictable bounce angles, depending on condition. During cooperative game play, participants reduced the standard deviation of their paddle hits with the ball and also reduced the absolute difference in distance between paddle hits from paddle center.

There was a significant effect of social context (cooperation or competition) on the performance of and strategy employed by the participants, evidenced by control of paddle kinematics. They also modified their ball contact strategy between conditions. We quantified the ball-to-paddle contact as a portion of the paddle height, where higher values reflected ball contact towards the top of the paddle. The game program was written such that, when the ball contacts portions of the paddle that are progressively further away from the center, it exits the paddle at a more acute angle across the screen. This increases vertical displacement and reduces trajectory predictability, as the ball may bounce off the top or bottom of the screen. Keeping the ball contact more towards the center of the paddle in the cooperative condition causes the ball to exit the paddle straighter across the screen, increasing the partner’s predictability of the ball motion.

These behavioral results contribute to an area of video game research that has thus far yielded inconclusive results. A number of studies using video games to assess prosocial behaviors found that cooperative or prosocial video game play influences subsequent prosocial behaviors after game play, as well as increases helpful behaviors and reduces hurtful behaviors [6, 7, 13]. However, more recent work in the field contrasts with these assertions. In Verheijen and colleague’s 2019 study, pairs of adolescent friends played a racing game in solitary, competitive, and cooperative conditions, after which their friendship quality and prosocial behavior towards the friend were measured. Results found that cooperative modes of game play increased both positive and negative behaviors towards friends. Furthermore, the authors found no effect of cooperative game play on subsequent prosocial behaviors [14]. These studies could be confounded by the presence of existing relationships between the dyads [14], or by the comparison performed in many studies using violent video games rather than strictly adjusting the social context of the game.

Here, we manipulated the social context of Pong game play by changing instructions to establish cooperative or competitive goals for each pair of participants within the same game structure and rules. It is well understood that the wording and delivery of instructions alone can direct participants toward specific tasks, movements, and motivations in motor skill learning and performance (for a review, see Wulf et al., 2010) [76]. Consequently, we changed only the instructions and left all other game features the same between conditions.

### 4.3 Limitations

The results of this study should be considered with the following limitations in mind. First, this study has a smaller sample size compared to some previous social psychology experiments which examined associations between prosocial tendencies and other behaviors [16, 29]. However, within the context of motor kinematic intention studies [39, 77] and the assessment of individual differences between cooperative and competitive game play, the sample size is more consistent with the existing literature. While we did identify multiple associations between survey dimensions and performance differential across conditions, some trends may have been enhanced by testing a larger number of participants.

Another limitation to this study is the method of incentivizing used to motivate participants to perform well during cooperative games. Not all participants were familiar with the original Pong game by name–the video is offered as an additional visual cue to stimulate their memory to avoid confusion with other games. However, to motivate performance for each game context, participant pairs were told that their performance for cooperative and competitive games would be recorded and ranked alongside other pairs of players. This added a level of competition to the cooperative games, which may mediate the cooperative context created by the researchers. Subjects also did not receive additional monetary incentives based on game outcomes in case it would moderate the cooperative/competitive contexts and player behavior.

The research team also removed three items of the revised PSB (PSB-30) that loaded onto the dimension “personal distress”. During piloting for the experiment, these items were confusing to participants and were removed from the PSB-30 to reduce the potential for misinterpretation of the items to impact the overall survey score. In hindsight, the responses to these items may have allowed for greater variation in participant responses for the overall survey, and also could have been used to calculate the factor “Helpfulness” onto which the dimensions “self-reported altruism” and “personal distress” load [65].

Last, a factor that may have limited the interpretation of our results is that the paddle controlled by the participants moved at a fixed speed rather than with proportional velocity. This is partially related to the difference in controls used by the participants–- keyboard buttons were used instead of the classic paddle from the Pong game. It is possible that we might have identified more dynamic changes in strategy between interaction conditions if the participants could control the speed at which the paddle moved up and down the screen. Previous intention kinematics studies have evaluated the kinematics of arm-reaching during changing social interactions and have found a change in the amplitude of peak velocity and movement time of the arm between cooperative and competitive scenarios [39].

## 5. Conclusion

In summary, we studied the individual differences in motor behavior during a two-person video game under changing social contexts. We found that participants effectively updated in-game movement kinematics to emphasize/support cooperative or competitive interactions with a human partner.

## Supporting information

S1 Appendix Task instructions and participant surveys. Cooperative and competitive game instructions for the experimental session and surveys administered to participants.
(DOCX)

S1 File Spearman rank correlation tables. Spearman rank correlation tables between all behavioral outcome variables and CAPSS, MSOS, and PSB scales and dimensions.
(DOCX)

S2 File Additional statistical analyses. Descriptive statistics, statistical power analyses and statistical checks for assumptions of normality.
(DOCX)
