# Supplementary material for: Individual differences in cooperative and competitive play strategies
Source: PLoS One. 2023 Nov 9;18(11):e0293583. doi: 10.1371/journal.pone.0293583 (PMC10635547; doi:10.1371/journal.pone.0293583)
Supplement: S1 Appendix — Cooperative and competitive game instructions for the experimental session and surveys administered to participants. (DOCX) [file pone.0293583.s001.docx]

**S1 Appendix**

## Subject Instructions, Cooperative and Competitive games

**Cooperation Prompt:**

Please read the prompt below *before* you continue on to perform these trials:

For these trials, you will play 6 bouts of Pong with your partner, each 3 minutes in duration. **Use your dominant hand to control the two keys for moving your paddle**. Work together with your partner to keep the scores at the top of the screen as close to (0 – 0) as you can. The goal is to score as *few points* against your partner as possible; you will accomplish this by working more cooperatively with your partner.

The researcher will monitor performance on a separate screen. Refrain from speaking aloud to one another during the trials – do not speak words of encouragement, advice, or make other intentional vocalizations that may affect your performance or the performance of your partner. At the end of the trial, the researcher will inform you what your best performance was for all the games, which will be added to a scoreboard for the experiment after the session is complete. Wait for the researcher to tell you to start a new trial.

The game console will close when the trial is over.

**Competition Prompt:**

Please read the prompt below *before* you continue on to perform these trials:

For these trials, you will play 6 bouts of Pong with your opponent, each 3 minutes in duration. **Use one hand to control the two keys for moving your paddle.** You are competing to score *more points* against your opponent by bouncing the ball off of the wall behind their paddle. The scores at the top of the screen show each person’s score compared to their opponent; therefore, you earn more points by playing more competitively.

The researcher will monitor performance on a separate screen. Refrain from speaking aloud to one another during the trial – do not speak words of discouragement, advice, or make other intentional vocalizations that may affect your performance or the performance of your partner. At the end of the trials, one player will be declared the winner of the round, and their progress will be posted anonymously to a scoreboard after the session is complete. At the end of the trial, wait for the researcher to tell you to start a new trial.

The game console will close when the trial is over.

## Copies of participant surveys, completed at the end of the experimental session.

Compliant and Principled Sportspersonship Scale (CAPSS-24) – developed by Perry et al. [1]–[3]

**Compliant and Principled Sportspersonship Scale-24**

**Sex:** Male 🞏 Female 🞏 **Age:**

**Main Sport Involved with: Years involved in main sport:**

**Current level of participation (please tick):**

**Recreational 🞏 Club 🞏 Semi-Professional 🞏 Professional 🞏**

Play in local leagues Play for established club Compete at national level Full-time

Play with friends Compete in regional leagues Receive payment for performance* Highest level in sport

Play for fun *in some sports

Instructions

Please read each statement below and for each indicate the extent to which firstly in section 1, you believe how you *ought* to behave. That is, do you believe that this is a good behaviour or attitude? You should indicate this by circling the appropriate number between 1 ‘Strongly Disagree’ and 5 ‘Strongly Agree’.

Secondly, indicate the extent to which you *actually* behave in section 2. Again, this should be indicated by circling the appropriate number between 1 ‘Strongly Disagree’ and 5 ‘Strongly Agree’.

Please answer all questions honestly.

|  | **Strongly Disagree** | **Disagree** | **Neutral** | **Agree** | **Strongly Agree** |
| --- | --- | --- | --- | --- | --- |
| 1. I never break the rules of my sport | 1 | 2 | 3 | 4 | 5 |
| 1. I never argue with a referring decision even if I feel it is wrong | 1 | 2 | 3 | 4 | 5 |
| 1. I do not believe in winning at all costs | 1 | 2 | 3 | 4 | 5 |
| 1. I abide by all of the rules in my sport | 1 | 2 | 3 | 4 | 5 |
| 1. I will always congratulate my opponent on his or her victory | 1 | 2 | 3 | 4 | 5 |
| 1. I would rather be respected for my actions than merely winning | 1 | 2 | 3 | 4 | 5 |
| 1. I would not intentionally injure an opponent to gain advantage | 1 | 2 | 3 | 4 | 5 |
| 1. I never argue with officials | 1 | 2 | 3 | 4 | 5 |
| 1. I would not bend the rules to win | 1 | 2 | 3 | 4 | 5 |
| 1. It is more important to do what is right than to win | 1 | 2 | 3 | 4 | 5 |
| 1. At times I will acknowledge my opponents good play | 1 | 2 | 3 | 4 | 5 |
| 1. I refrain from tactics that could injure my opponent | 1 | 2 | 3 | 4 | 5 |

|  | **Strongly Disagree** | **Disagree** | **Neutral** | **Agree** | **Strongly Agree** |
| --- | --- | --- | --- | --- | --- |
| 1. I would rather lose with grace than win with dishonesty | 1 | 2 | 3 | 4 | 5 |
| 1. I never vent my frustrations on match officials | 1 | 2 | 3 | 4 | 5 |
| 1. I truly respect a worthy opponent | 1 | 2 | 3 | 4 | 5 |
| 1. I play hard but make sure that I do not injure my opponent | 1 | 2 | 3 | 4 | 5 |
| 1. I do not swear at officials | 1 | 2 | 3 | 4 | 5 |
| 1. Winning is not always the most important part of sport | 1 | 2 | 3 | 4 | 5 |
| 1. It is wrong to test the boundaries to see what I can get away with | 1 | 2 | 3 | 4 | 5 |
| 1. I would go out of my way to congratulate an opponent | 1 | 2 | 3 | 4 | 5 |
| 1. I would never intentionally foul an opponent | 1 | 2 | 3 | 4 | 5 |
| 1. The official’s decision is final and I accept that | 1 | 2 | 3 | 4 | 5 |
| 1. I always obey the rules of my sport | 1 | 2 | 3 | 4 | 5 |
| 1. It is more important to play fair than to win | 1 | 2 | 3 | 4 | 5 |

Thank you for your participation.

Multidimensional Sportspersonship Orientation Scale (MSOS-25) – developed and validated by Vallerand et al [4].

**Behavior in Sport**

Indicate which sport you refer to while answering the next 25 questions: _______________________

For each of the following items, circle the number that best represents the extent to which the item corresponds to you with respect to the sport you identified above.

| **Doesn’t correspond to me at all** | **Corresponds to me a little** | **Corresponds to me partly** | **Corresponds to me a lot** | **Corresponds to me exactly** |
| --- | --- | --- | --- | --- |
| **1** | **2** | **3** | **4** | **5** |

| 1. When I lose, I congratulate the opponent whoever he or she is. | 1 | 2 | 3 | 4 | 5 | N/A |
| --- | --- | --- | --- | --- | --- | --- |
| 1. I obey the referee. | 1 | 2 | 3 | 4 | 5 | N/A |
| 1. In competition, I go all out even if I’m almost sure to lose. | 1 | 2 | 3 | 4 | 5 | N/A |
| 1. I help the opponent get up after a fall. | 1 | 2 | 3 | 4 | 5 | N/A |
| 1. I compete for personal honors, trophies, and medals. | 1 | 2 | 3 | 4 | 5 | N/A |
| 1. After a defeat, I shake hands with the opponent’s coach. | 1 | 2 | 3 | 4 | 5 | N/A |
| 1. I respect the rules. | 1 | 2 | 3 | 4 | 5 | N/A |
| 1. I don’t give up even after making many mistakes. | 1 | 2 | 3 | 4 | 5 | N/A |
| 1. If I can, I ask the referee to allow the opponent who has been unjustly disqualified to keep on playing. | 1 | 2 | 3 | 4 | 5 | N/A |
| 1. I criticize what the coach makes me do. | 1 | 2 | 3 | 4 | 5 | N/A |
| 1. After a competition, I congratulate the opponent for his good performance. | 1 | 2 | 3 | 4 | 5 | N/A |
| 1. I really obey all rules of my sport. | 1 | 2 | 3 | 4 | 5 | N/A |
| 1. I think about ways to improve my weaknesses. | 1 | 2 | 3 | 4 | 5 | N/A |
| 1. When an opponent gets hurt, I ask the referee to stop the game so that he or she can get help. | 1 | 2 | 3 | 4 | 5 | N/A |
| 1. After a competition, I use excuses for a bad performance. | 1 | 2 | 3 | 4 | 5 | N/A |
| 1. After a win, I acknowledge the opponent’s good work. | 1 | 2 | 3 | 4 | 5 | N/A |
| 1. I respect the referee even when he or she is not good. | 1 | 2 | 3 | 4 | 5 | N/A |
| 1. It is important to me to be present at all practices. | 1 | 2 | 3 | 4 | 5 | N/A |
| 1. If I see that the opponent is unjustly penalized, I try to rectify the situation. | 1 | 2 | 3 | 4 | 5 | N/A |
| 1. When my coach points out my mistakes after a competition, I refuse to admit that I made those mistakes. | 1 | 2 | 3 | 4 | 5 | N/A |
| 1. Win or lose, I shake hands with the opponent after the game. | 1 | 2 | 3 | 4 | 5 | N/A |
| 1. I respect an official’s decision even if he or she is not the referee. | 1 | 2 | 3 | 4 | 5 | N/A |
| 1. During practices, I go all out. | 1 | 2 | 3 | 4 | 5 | N/A |
| 1. If by misfortune, an opponent forgets his or her equipment, I lend him my spare one. | 1 | 2 | 3 | 4 | 5 | N/A |
| 1. If I make a mistake during a crucial time of the match, I get angry. | 1 | 2 | 3 | 4 | 5 | N/A |

Penner Prosocial Battery, revised 30 (PSB-27) – original survey developed and validated by Louis Penner [5].

Penner Prosocial Personality Battery (PSB-30 revised)

**PART 1:**

Below are a number of statements which may or may not describe you, your feelings or your behavior. Please read each statement carefully and blacken in the space on your answer sheet which corresponds to choices presented below. There are no right or wrong responses.

Use the following scale to indicate your answer:

| **Strongly Disagree** | **Disagree** | **Uncertain** | **Agree** | **Strongly Agree** |
| --- | --- | --- | --- | --- |
| **1** | **2** | **3** | **4** | **5** |

1. When people are nasty to me, I feel very little responsibility to treat them well. _______
2. I would feel less bothered about leaving litter in a dirty park than in a clean one. _______
3. No matter what a person has done to us, there is no excuse for taking advantage of them. _______
4. With the pressure for grades and the widespread cheating in school nowadays, the individual who cheats occasionally is not really as much at fault. _______
5. It doesn't make much sense to be very concerned about how we act when we are sick and feeling miserable. _______
6. If I broke a machine through mishandling, I would feel less guilty if it was already damaged before I used it. _______
7. When you have a job to do, it is impossible to look out for everybody’s best interest. _______
8. I sometimes find it difficult to see things from the "other person's" point of view. _______
9. When I see someone being taken advantage of, I feel kind of protective towards them. _______
10. I sometimes try to understand my friends better by imagining how things look from their perspective. _______
11. Other people's misfortunes do not usually disturb me a great deal. _______
12. If I'm sure I'm right about something, I don't waste much time listening to other people's arguments. _______
13. When I see someone being treated unfairly, I sometimes don’t feel very much pity for them. _______
14. I am often quite touched by things that I see happen. _______
15. I believe that there are two sides to every question and try to look at them both. _______
16. When I'm upset at someone, I usually try to "put myself in their shoes" for a while. _______

**PART 2:**

Below are a set of statements which may or may not describe how you make decisions when you

have to choose between two courses of action or alternatives when there is no clear right way or

wrong way to act. Some examples of such situations are: being asked to lend something to a

close friend who often forgets to return things; deciding whether you should keep something you

have won for yourself or share it with a friend; and choosing between studying for an important

exam and visiting a sick relative. Read each statement and blacken in the space on your answer

sheet which corresponds to the choices presented below.

──────────────────────────────────────────────────────

| **Strongly Disagree** | **Disagree** | **Uncertain** | **Agree** | **Strongly Agree** |
| --- | --- | --- | --- | --- |
| **1** | **2** | **3** | **4** | **5** |

──────────────────────────────────────────────────────

1. My decisions are usually based on my concern for other people. _______
2. My decisions are usually based on what is the most fair and just way to act. _______
3. I choose alternatives that are intended to meet everybody's needs. _______
4. I choose a course of action that maximizes the help other people receive. _______
5. I choose a course of action that considers the rights of all people involved. _______
6. My decisions are usually based on concern for the welfare of others. _______

**PART 3:**

Below are several different actions in which people sometimes engage. Read each of them and

decide how frequently you have carried it out in the past. Blacken in the space on your answer

sheet which best describes your past behavior. Use the scale presented below.

─────────────────────────────────────────────────────

| **Never** | **Once** | **More than Once** | **Often** | **Very Often** |
| --- | --- | --- | --- | --- |
| **1** | **2** | **3** | **4** | **5** |

──────────────────────────────────────────────────────

1. I have helped carry a stranger's belongings (e.g., books, parcels, etc.). _______
2. I have allowed someone to go ahead of me in a line (e.g., supermarket, copying machine, etc.). _______
3. I have let a neighbor whom I didn't know too well borrow an item of some value (e.g., tools, a dish, etc.). _______
4. I have, before being asked, voluntarily looked after a neighbor's pets or children without being paid for it. _______
5. I have offered to help a handicapped or elderly stranger across a street. _______

References:

[1] J. L. Perry and P. J. Clough, “Predicting cooperation in competitive conditions: The role of sportspersonship, moral competence, and emotional intelligence,” *Psychol. Sport Exerc.*, vol. 31, pp. 88–92, Jul. 2017, doi: 10.1016/j.psychsport.2017.04.008.

[2] J. L. Perry, P. J. Clough, L. Crust, S. L. Nabb, and A. R. Nicholls, “Development and validation of the compliant and principled sportspersonship scale,” *Res. Q. Exerc. Sport*, vol. 86, no. 1, pp. 71–80, 2015, doi: 10.1080/02701367.2014.980938.

[3] J. L. Perry, “Redefining Sportspersonship : A Compliant and Principled Model,” 2014.

[4] P. Vallerand, R.J, Brière, N. M., Blanchard, C., & Provencher, “Development and validation of the Multidimensional Sportspersonship Orientations Scale. - PsycNET,” *J. Sport Exerc. Psychol.*, 1997, Accessed: Sep. 29, 2020. [Online]. Available: https://psycnet.apa.org/record/1997-04763-007

[5] L. A. Penner, B. A. Fritzche, J. P. Craiger, and T. S. Freifeld, “Measuring The Prosocial Personality,” *Adv. Personal. Assess.*, vol. 10, no. January 1995, p. 209, 1995.
