# Supplementary material for: Individual differences in cooperative and competitive play strategies
Source: PLoS One. 2023 Nov 9;18(11):e0293583. doi: 10.1371/journal.pone.0293583 (PMC10635547; doi:10.1371/journal.pone.0293583)
Supplement: S1 File — Spearman rank correlation tables between all behavioral outcome variables and CAPSS, MSOS, and PSB scales and dimensions. (DOCX) [file pone.0293583.s002.docx]

S2 Supporting Information. Full results of Spearman rank correlations between personality surveys/ survey subdimensions and behavioral outcomes.

# CAPSS survey and dimensions

**Table A.** All spearman correlations of outcome variables and total CAPSS survey score.

| **Outcome** | **Spearman's rho (ρ)** | **p-value** | **Adjusted p-value (B-H)** |
| --- | --- | --- | --- |
| Average Points, cooperation | .29 | 0.067 | 0.543 |
| PP_SD_, competition | -.28 | 0.081 | 0.543 |
| Average Points, all trials | .22 | 0.172 | 0.543 |
| PP_diffABS_, competition | -.19 | 0.251 | 0.543 |
| PP_mean_, competition | .18 | 0.2697 | 0.543 |
| PP_SD_, all trials | -.17 | 0.3071 | 0.543 |
| PP_diffABS_, cooperation | .16 | 0.3149 | 0.562 |
| Average Points, competition | .15 | 0.3471 | 0.622 |
| PP_mean_, all trials | .11 | 0.4828 | 0.622 |
| PP_diffABS_, all trials | -.05 | 0.7496 | 0.623 |
| PP_SD_, cooperation | .03 | 0.8629 | 0.623 |
| PP_mean_, cooperation | 3.6E-03 | 0.9825 | 0.640 |

*All trials = averaged across all trials for participant, cooperation = average value of cooperation trial performance; competition = average value of competition trial performance. Bolded p-values indicate significance.*

**Table B**. Results of all outcome variables and the CAPSS dimension “game perspective”.

| **Outcome** | **Spearman's rho (ρ)** | **p-value** | **Adjusted p-value (B-H)** |
| --- | --- | --- | --- |
| Average Points, cooperation | .36 | **0.023*** | 0.272 |
| Average Points, all trials | .31 | 0.052 | 0.311 |
| Average Points, competition | .23 | 0.146 | 0.565 |
| PP_SD_, competition | -.21 | 0.188 | 0.565 |
| PP_diffABS_, cooperation | .16 | 0.313 | 0.609 |
| PP­_SD_, all trials | -.16 | 0.335 | 0.609 |
| PP_mean_, all trials | -.15 | 0.355 | 0.609 |
| PP_mean_, cooperation | -.12 | 0.457 | 0.685 |
| PP_mean_, competition | -.06 | 0.722 | 0.946 |
| PP_diffABS_, competition | -.04 | 0.788 | 0.946 |
| PP_diffABS_, all trials | .01 | 0.939 | 0.990 |
| PP_SD_, cooperation | -2.1E-03 | 0.990 | 0.990 |

*All trials = averaged across all trials for participant, cooperation = average value of cooperation trial performance; competition = average value of competition trial performance. Bolded p-values indicate significance.*

**Table C.** Results of all outcome variables and the CAPSS dimension “legitimizing injurious acts”.

| **Outcome** | **Spearman's rho (ρ)** | **p-value** | **Adjusted p-value (B-H)** |
| --- | --- | --- | --- |
| PP_SD_, competition | -.38 | **0.015** | 0.075 |
| PP_diffABS_, competition | -.37 | **0.018*** | 0.075 |
| Average Points, cooperation | .37 | **0.019*** | 0.075 |
| Average Points, both conditions | .33 | **0.036*** | 0.106 |
| PP_mean_, all trials | .28 | 0.081 | 0.192 |
| PP_mean_, competition | .26 | 0.100 | 0.192 |
| Average Points, competition | .26 | 0.112 | 0.192 |
| PP­_SD_, all trials | -.24 | 0.140 | 0.209 |
| PP_diffABS_, cooperation | .30 | 0.170 | 0.226 |
| PP_mean_, cooperation | .20 | 0.224 | 0.268 |
| PP_diffABS_, all trials | -.14 | 0.398 | 0.434 |
| PP_SD_, cooperation | .02 | 0.923 | 0.923 |

*All trials = averaged across all trials for participant, cooperation = average value of cooperation trial performance; competition = average value of competition trial performance. Bolded p-values indicate significance.*

**Table D***.* Results of all outcome variables and the CAPSS dimension “compliance with officials”.

| **Outcome** | **Spearman's rho (ρ)** | **p-value** | **Adjusted p-value (B-H)** |
| --- | --- | --- | --- |
| PP_diffABS_, cooperation | .24 | 0.139 | 0.570 |
| PP_SD_, cooperation | .19 | 0.233 | 0.570 |
| PP_SD_, competition | -.19 | 0.254 | 0.570 |
| Average Points, competition | .16 | 0.321 | 0.570 |
| PP_diffABS_, competition | -.16 | 0.339 | 0.570 |
| Average Points, cooperation | .15 | 0.353 | 0.570 |
| Average Points, all trials | .15 | 0.370 | 0.570 |
| PP_mean_, all trials | .14 | 0.380 | 0.570 |
| PP_mean_, competition | .12 | 0.447 | 0.600 |
| PP_diffABS_, all trials | .07 | 0.654 | 0.785 |
| PP_mean_, cooperation | .03 | 0.834 | 0.910 |
| PP­_SD_, all trials | 7.7E-03 | 0.962 | 0.962 |

*All trials = averaged across all trials for participant, cooperation = average value of cooperation trial performance; competition = average value of competition trial performance. Bolded p-values indicate significance.*

**Table E**. Results of all outcome variables and the CAPSS dimension Respect for Opponents.

| **Outcome** | **Spearman's rho (ρ)** | **p-value** | **Adjusted p-value (B-H)** |
| --- | --- | --- | --- |
| PP_diffABS_, cooperation | -.31 | 0.051 | 0.616 |
| PP_SD_, cooperation | -.22 | 0.166 | 0.818 |
| PP_diffABS_, all trials | -.18 | 0.261 | 0.818 |
| PP­_SD_, all trials | -.12 | 0.454 | 0.818 |
| Average Points, competition | -.12 | 0.460 | 0.818 |
| Average Points, all trials | -.12 | 0.460 | 0.818 |
| PP_mean_, competition | .10 | 0.558 | 0.818 |
| Average Points, cooperation | -.09 | 0.593 | 0.818 |
| PP_mean_, all trials | .07 | 0.673 | 0.818 |
| PP_mean_, cooperation | .05 | 0.743 | 0.818 |
| PP_SD_, competition | .04 | 0.816 | 0.818 |
| PP_diffABS_, competition | .04 | 0.818 | 0.818 |

*All trials = averaged across all trials for participant, cooperation = average value of cooperation trial performance; competition = average value of competition trial performance. Bolded p-values indicate significance.*

**Table F**. Results of all outcome variables and the CAPSS dimension “compliance with rules”.

| **Outcome** | **Spearman's rho (ρ)** | **p-value** | **Adjusted p-value (B-H)** |
| --- | --- | --- | --- |
| Average Points, cooperation | .20 | 0.213 | 0.803 |
| PP_diffABS_, cooperation | .17 | 0.283 | 0.803 |
| PP_SD_, competition | -.17 | 0.296 | 0.803 |
| PP_diffABS_, competition | -.15 | 0.360 | 0.803 |
| Average Points, all trials | .14 | 0.393 | 0.803 |
| PP_mean_, competition | .13 | 0.426 | 0.803 |
| PP­_SD_, all trials | -.11 | 0.506 | 0.803 |
| Average Points, competition | .10 | 0.553 | 0.803 |
| PP_mean_, all trials | .08 | 0.605 | 0.803 |
| PP_mean_, cooperation | .07 | 0.669 | 0.803 |
| PP_SD_, cooperation | .06 | 0.736 | 0.803 |
| PP_diffABS_, all trials | -.02 | 0.915 | 0.915 |

*All trials = averaged across all trials for participant, cooperation = average value of cooperation trial performance; competition = average value of competition trial performance. Bolded p-values indicate significance.*

# MSOS survey and dimensions

**Table G**. Results of all outcome variables and the total MSOS survey score.

| **Outcome** | **Spearman's rho (ρ)** | **p-value** | **Adjusted p-value (B-H)** |
| --- | --- | --- | --- |
| PP_diffABS_, cooperation | -.27 | 0.098 | 0.543 |
| Average Points, cooperation | -.26 | 0.102 | 0.543 |
| Average Points, all trials | -.23 | 0.151 | 0.543 |
| PP_SD_, cooperation | -.22 | 0.184 | 0.543 |
| Average Points, competition | -.19 | 0.252 | 0.543 |
| PP­_SD_, all trials | -.18 | 0.272 | 0.543 |
| PP_SD_, competition | -.16 | 0.328 | 0.562 |
| PP_mean_, competition | .13 | 0.419 | 0.622 |
| PP_mean_, all trials | -.11 | 0.518 | 0.622 |
| PP_mean_, cooperation | -.10 | 0.538 | 0.622 |
| PP_diffABS_, competition | -.09 | 0.571 | 0.622 |
| PP_diffABS_, all trials | -.08 | 0.640 | 0.640 |

*All trials = averaged across all trials for participant, cooperation = average value of cooperation trial performance; competition = average value of competition trial performance. Bolded p-values indicate significance.*

**Table H.** Results of all outcome variables and the MSOS dimension “respect for full commitment to sport participation”.

| **Outcome** | **Spearman's rho (ρ)** | **p-value** | **Adjusted p-value (B-H)** |
| --- | --- | --- | --- |
| PP_mean_, all trials | .24 | 0.139 | 0.890 |
| PP_mean_, cooperation | .16 | 0.310 | 0.890 |
| Average Points, all trials | -.11 | 0.497 | 0.890 |
| Average Points, competition | -.10 | 0.531 | 0.890 |
| Average Points, cooperation | -.10 | 0.556 | 0.890 |
| PP_mean_, competition | .10 | 0.558 | 0.890 |
| PP_diffABS_, competition | .08 | 0.609 | 0.890 |
| PP_SD_, cooperation | .07 | 0.651 | 0.890 |
| PP­_SD_, all trials | .07 | 0.668 | 0.890 |
| PP_diffABS_, all trials | .03 | 0.836 | 0.946 |
| PP_SD_, competition | .02 | 0.897 | 0.946 |
| PP_diffABS_, cooperation | -.01 | 0.946 | 0.946 |

*All trials = averaged across all trials for participant, cooperation = average value of cooperation trial performance; competition = average value of competition trial performance. Bolded p-values indicate significance.*

**Table I.** Results of Spearman correlations between all outcome variables and the MSOS dimension “negative approach to sport participation”.

| **Outcome** | **Spearman's rho (ρ)** | **p-value** | **Adjusted p-value (B-H)** |
| --- | --- | --- | --- |
| PP_diffABS_, all trials | .27 | 0.092 | 0.538 |
| PP_diffABS_, competition | .27 | 0.096 | 0.537 |
| Average Points, competition | .20 | 0.207 | 0.537 |
| PP_diffABS_, cooperation | .20 | 0.211 | 0.537 |
| PP_SD_, cooperation | .19 | 0.235 | 0.537 |
| PP­_SD_, all trials | .16 | 0.333 | 0.537 |
| Average Points, all trials | .15 | 0.348 | 0.537 |
| PP_mean_, competition | -.15 | 0.363 | 0.537 |
| PP_mean_, all trials | -.14 | 0.403 | 0.537 |
| Average Points, cooperation | .09 | 0.599 | 0.671 |
| PP_SD_, competition | .08 | 0.615 | 0.671 |
| PP_mean_, cooperation | -.04 | 0.8003 | 0.800 |

*All trials = averaged across all trials for participant, cooperation = average value of cooperation trial performance; competition = average value of competition trial performance. Bolded p-values indicate significance.*

**Table J**. Results of all outcome variables and the MSOS dimension “respect and concern for opponents”.

| **Outcome** | **Spearman's rho (ρ)** | **p-value** | **Adjusted p-value (B-H)** |
| --- | --- | --- | --- |
| PP_mean_, competition | -.23 | 0.146 | 0.507 |
| PP_mean_, all trials | -.22 | 0.181 | 0.507 |
| PP_SD_, competition | -.20 | 0.187 | 0.507 |
| PP­_SD_, all trials | -.21 | 0.1887 | 0.507 |
| PP_SD_, cooperation | -.18 | 0.258 | 0.507 |
| PP_diffABS_, cooperation | -.17 | 0.292 | 0.507 |
| PP_mean_, cooperation | -.15 | 0.366 | 0.507 |
| Average Points, all trials | -.14 | 0.406 | 0.507 |
| Average Points, cooperation | -.13 | 0.412 | 0.507 |
| Average Points, competition | -.13 | 0.423 | 0.507 |
| PP_diffABS_, all trials | -.06 | 0.722 | 0.770 |
| PP_diffABS_, competition | .05 | 0.770 | 0.770 |

*All trials = averaged across all trials for participant, cooperation = average value of cooperation trial performance; competition = average value of competition trial performance. Bolded p-values indicate significance.*

**Table K**. Results of all outcome variables and the MSOS dimension “respect for rules and officials”.

| **Outcome** | **Spearman's rho (ρ)** | **p-value** | **Adjusted p-value (B-H)** |
| --- | --- | --- | --- |
| PP_mean_, competition | .14 | 0.392 | 0.962 |
| Average Points, competition | -.13 | 0.409 | 0.962 |
| PP_mean_, cooperation | -.12 | 0.465 | 0.962 |
| Average Points, all trials | -.07 | 0.685 | 0.962 |
| PP_diffABS_, competition | -.05 | 0.774 | 0.962 |
| PP_diffABS_, cooperation | .04 | 0.812 | 0.962 |
| PP_SD_, competition | -.04 | 0.818 | 0.962 |
| PP_SD_, cooperation | .04 | 0.825 | 0.962 |
| Average Points, cooperation | -.03 | 0.836 | 0.962 |
| PP_mean_, all trials | .03 | 0.838 | 0.962 |
| PP­_SD_, all trials | -.02 | 0.925 | 0.962 |
| PP_diffABS_, all trials | -.01 | 0.962 | 0.962 |

*All trials = averaged across all trials for participant, cooperation = average value of cooperation trial performance; competition = average value of competition trial performance. Bolded p-values indicate significance.*

**Table L**. Results of all outcome variables and the MSOS dimension “respect for social convention”.

| **Outcome** | **Spearman's rho (ρ)** | **p-value** | **Adjusted p-value (B-H)** |
| --- | --- | --- | --- |
| Average Points, cooperation | -.41 | **0.008**** | 0.095 |
| Average Points, all trials | -.37 | **0.020*** | 0.118 |
| Average Points, competition | -.27 | 0.098 | 0.391 |
| PP_diffABS_, cooperation | -.22 | 0.181 | 0.483 |
| PP_diffABS_, competition | .21 | 0.201 | 0.483 |
| PP_SD_, cooperation | -.12 | 0.478 | 0.926 |
| PP_mean_, competition | -.07 | 0.672 | 0.926 |
| PP_mean_, all trials | -.07 | 0.678 | 0.926 |
| PP_SD_, competition | .05 | 0.739 | 0.926 |
| PP_mean_, cooperation | -.05 | 0.772 | 0.926 |
| PP­_SD_, all trials | .012 | 0.943 | 0.989 |
| PP_diffABS_, all trials | 2.4E-03 | 0.989 | 0.989 |

*All trials = averaged across all trials for participant, cooperation = average value of cooperation trial performance; competition = average value of competition trial performance. Bolded p-values indicate significance.*

# PSB survey and dimensions

**Table M**. Results of all outcome variables and the PSB survey score.

| **Outcome** | **Spearman's rho (ρ)** | **p-value** | **Adjusted p-value (B-H)** |
| --- | --- | --- | --- |
| PP_diffABS_, competition | .18 | 0.256 | 0.908 |
| PP_diffABS_, all trials | .17 | 0.303 | 0.908 |
| Average Points, competition | .17 | 0.306 | 0.908 |
| PP_SD_, cooperation | .14 | 0.402 | 0.908 |
| Average Points, all trials | .13 | 0.428 | 0.908 |
| PP_diffABS_, cooperation | .12 | 0.457 | 0.908 |
| PP­_SD_, all trials | .09 | 0.580 | 0.908 |
| PP_mean_, cooperation | .08 | 0.646 | 0.908 |
| Average Points, cooperation | .07 | 0.686 | 0.908 |
| PP_SD_, competition | .05 | 0.774 | 0.908 |
| PP_mean_, competition | .03 | 0.833 | 0.908 |
| PP_mean_, both conditions | .02 | 0.913 | 0.913 |

*All trials = averaged across all trials for participant, cooperation = average value of cooperation trial performance; competition = average value of competition trial performance. Bolded p-values indicate significance.*

**Table N**. Results of all Spearman correlations between outcome variables and the PSB dimension “empathic concern”.

| **Outcome** | **Spearman’s rho (ρ)** | **p-value** | **Adjusted p-value (B-H)** |
| --- | --- | --- | --- |
| PP_diffABS_, cooperation | .48 | **0.002**** | **0.022*** |
| PP_SD_, cooperation | .45 | **0.004**** | **0.022*** |
| PP_diffABS_, all trials | .35 | **0.025*** | 0.097 |
| Average Points, cooperation | .34 | **0.032*** | 0.097 |
| Average Points, all trials | .31 | 0.051 | 0.122 |
| PP­_SD_, all trials | .28 | 0.081 | 0.162 |
| Average Points, competition | .27 | 0.095 | 0.163 |
| PP_mean_, competition | .18 | 0.273 | 0.410 |
| PP_mean_, cooperation | -.12 | 0.459 | 0.565 |
| PP_SD_, competition | .12 | 0.471 | 0.565 |
| PP_diffABS_, competition | .05 | 0.757 | 0.825 |
| PP_mean_, all trials | -.02 | 0.918 | 0.918 |

*All trials = averaged across all trials for participant, cooperation = average value of cooperation trial performance; competition = average value of competition trial performance. Bolded p-values indicate significance.*

**Table O**. Results of all outcome variables and the PSB dimension “moral reasoning”.

| **Outcome** | **Spearman's rho (ρ)** | **p-value** | **Adjusted p-value (B-H)** |
| --- | --- | --- | --- |
| Average Points, competition | .22 | 0.164 | 0.872 |
| Average Points, all trials | .22 | 0.182 | 0.872 |
| Average Points, cooperation | .16 | 0.312 | 0.872 |
| PP_mean_, all trials | .16 | 0.313 | 0.872 |
| PP_mean_, competition | .14 | 0.386 | 0.872 |
| PP_mean_, cooperation | .13 | 0.442 | 0.872 |
| PP_diffABS_, competition | .11 | 0.509 | 0.872 |
| PP_diffABS_, all trials | .06 | 0.736 | 0.885 |
| PP­_SD_, all trials | .05 | 0.756 | 0.885 |
| PP_SD_, cooperation | .03 | 0.852 | 0.885 |
| PP_SD_, competition | .03 | 0.879 | 0.885 |
| PP_diffABS_, cooperation | .02 | 0.885 | 0.885 |

*All trials = averaged across all trials for participant, cooperation = average value of cooperation trial performance; competition = average value of competition trial performance. Bolded p-values indicate significance.*

**Table P**. Results of all outcome variables and the PSB dimension “perspective taking”.

| **Outcome** | **Spearman's rho (ρ)** | **p-value** | **Adjusted p-value (B-H)** |
| --- | --- | --- | --- |
| PP_diffABS_, competition | .24 | 0.140 | 0.959 |
| PP_diffABS_, all trials | .15 | 0.36 | 0.959 |
| Average Points, competition | .13 | 0.410 | 0.959 |
| PP_SD_, competition | .13 | 0.414 | 0.959 |
| PP­_SD_, all trials | .09 | 0.579 | 0.959 |
| PP_diffABS_, cooperation | .09 | 0.602 | 0.959 |
| PP_mean_, competition | -.06 | 0.697 | 0.959 |
| Average Points, all trials | .05 | 0.763 | 0.959 |
| PP_SD_, cooperation | .04 | 0.815 | 0.959 |
| PP_mean_, cooperation | .03 | 0.830 | 0.959 |
| Average Points, cooperation | -.03 | 0.879 | 0.959 |
| PP_mean_, all trials | -.01 | 0.959 | 0.959 |

*All trials = averaged across all trials for participant, cooperation = average value of cooperation trial performance; competition = average value of competition trial performance. Bolded p-values indicate significance.*

**Table Q**. Results of all outcome variables and the MSOS dimension “social responsibility”.

| **Outcome** | **Spearman's rho (ρ)** | **p-value** | **Adjusted p-value (B-H)** |
| --- | --- | --- | --- |
| PP_diffABS_, competition | .24 | 0.135 | 0.986 |
| PP_mean_, competition | -.22 | 0.175 | 0.986 |
| PP_mean_, cooperation | .17 | 0.297 | 0.986 |
| PP_mean_, all trials | -.11 | 0.513 | 0.986 |
| Average Points, competition | .09 | 0.589 | 0.986 |
| PP_diffABS_, cooperation | -.09 | 0.589 | 0.986 |
| PP_diffABS_, all trials | .07 | 0.690 | 0.986 |
| Average Points, all trials | .06 | 0.721 | 0.986 |
| PP_SD_, competition | .03 | 0.843 | 0.986 |
| PP_SD_, cooperation | -.01 | 0.939 | 0.986 |
| PP­_SD_, all trials | -.01 | 0.952 | 0.986 |
| Average Points, cooperation | 2.8E-03 | 0.986 | 0.986 |

*All trials = averaged across all trials for participant, cooperation = average value of cooperation trial performance; competition = average value of competition trial performance. Bolded p-values indicate significance.*

**Table R**. Results of all outcome variables and the PSB dimension “self-reported altruism”.

| **Outcome** | **Spearman's rho (ρ)** | **p-value** | **Adjusted p-value (B-H)** |
| --- | --- | --- | --- |
| PP_SD_, competition | -.11 | 0.483 | 0.950 |
| PP_diffABS_, all trials | .10 | 0.544 | 0.950 |
| PP_mean_, competition | .09 | 0.562 | 0.950 |
| PP_diffABS_, cooperation | .09 | 0.575 | 0.950 |
| PP_SD_, cooperation | .09 | 0.586 | 0.950 |
| Average Points, cooperation | -.08 | 0.608 | 0.950 |
| Average Points, all trials | -.04 | 0.811 | 0.950 |
| PP_mean_, cooperation | -.03 | 0.853 | 0.950 |
| PP_diffABS_, competition | .03 | 0.870 | 0.950 |
| Average Points, competition | -.01 | 0.937 | 0.950 |
| PP_mean_, all trials | .01 | 0.949 | 0.950 |
| PP­_SD_, all trials | -.01 | 0.950 | 0.950 |

*All trials = averaged across all trials for participant, cooperation = average value of cooperation trial performance; competition = average value of competition trial performance. Bolded p-values indicate significance.*
