# Supplementary material for: Individual differences in cooperative and competitive play strategies
Source: PLoS One. 2023 Nov 9;18(11):e0293583. doi: 10.1371/journal.pone.0293583 (PMC10635547; doi:10.1371/journal.pone.0293583)
Supplement: S2 File — Descriptive statistics, statistical power analyses and statistical checks for assumptions of normality. (DOCX) [file pone.0293583.s003.docx]

S3 File. Tables and results of statistical analyses of power and assumptions of normality.

**Table A.** Descriptive statistics of outcome and predictor variables, no condition grouping

| **Variable** | **Mean** | **SD** | **Median (25^th^, 75^th^ %ile)** | **Min** | **Max** | **Range** | **Skew** | **Kurt** | **(SE)** |
| --- | --- | --- | --- | --- | --- | --- | --- | --- | --- |
| Points | 3.58 | 2.22 | 3 (2, 5) | 0 | 13 | 13 | 0.88 | 1.1 | 0.1 |
| PP_mean_ | 44.3 | 4.03 | 44.63 (42.29, 46.94) | 21.56 | 61.88 | 40.32 | -0.61 | 2.55 | 0.18 |
| PP_SD_ | 21.87 | 7.3 | 20.09 (18.29, 22.51) | 13.34 | 63.3 | 49.96 | 2.72 | 8.57 | 0.33 |
| PP_diffABS_ | 17.54 | 3.56 | 17.38 (15.33, 19.34) | 9.79 | 35.08 | 25.29 | 0.71 | 1.5 | 0.16 |
| PSB | 97.7 | 9.17 | 95.5 (91, 105.5) | 81 | 114 | 33 | 0.05 | -1.06 | 0.42 |
| MSOS | 85 | 13.67 | 89.5 (74.5, 94.5) | 48 | 108 | 60 | -0.84 | -0.01 | 0.62 |
| CAPSS | 100.5 | 9.38 | (94, 107.25) | 77 | 120 | 43 | -0.29 | -0.52 | 0.43 |

*SD = standard deviation, Min = minimum, Max = maximum, Skew = skewness, Kurt = kurtosis, SE = standard error*

**Table B.** Descriptive statistics of outcome variables, separated by condition

| **Variable** | **Condition** | **Mean** | **SD** | **Med.** | **Min** | **Max** | **Range** | **Skew** | **Kurt** | **SE** |
| --- | --- | --- | --- | --- | --- | --- | --- | --- | --- | --- |
| Points | Cooperation | 3.254 | 2.149 | 3 | 0 | 11 | 11 | 0.956 | 0.787 | 0.139 |
|  | Competition | 3.904 | 2.245 | 4 | 0 | 13 | 13 | 0.817 | 1.419 | 0.145 |
| PP_mean_ | Cooperation | 0.451 | 0.034 | 0.452 | 0.348 | 0.619 | 0.271 | 0.184 | 2.771 | 0.002 |
|  | Competition | 0.434 | 0.045 | 0.438 | 0.215 | 0.522 | 0.307 | -0.74 | 1.623 | 0.003 |
| PP_SD_ | Cooperation | 0.202 | 0.064 | 0.1897 | 0.133 | 0.540 | 0.407 | 2.898 | 10.186 | 0.004 |
|  | Competition | 0.235 | 0.078 | 0.214 | 0.157 | 0.63322 | 0.476 | 2.672 | 7.503 | 0.005 |
| PP_ABS_ | Cooperation | 0.159 | 0.031 | 0.157 | 0.098 | 0.280 | 0.182 | 0.697 | 0.883 | 0.002 |
|  | Competition | 0.192 | 0.032 | 0.185 | 0.133 | 0.351 | 0.218 | 1.226 | 2.827 | 0.002 |

*SD = standard deviation, Med. = median Min = minimum, Max = maximum, Skew = skewness, Kurt = kurtosis, SE = standard error*

**Table C.** Descriptive statistics of comparison of residuals between original and rank-ordered variables in linear mixed effect model

| **Outcome Variable** | **Model data** | **Mean** | **SD** | **Med (25^th^, 75^th^ %ile)** | **Min** | **Max** | **Range** | **SE** |
| --- | --- | --- | --- | --- | --- | --- | --- | --- |
| Points | Original | 0 | 1.59 | -0.13 (-1.03, 0.91) | -4.28 | 5.46 | 9.74 | 0.07 |
|  | Rank ordered | 0 | 1.59 | -0.11 (-1.02, 0.90) | -4.25 | 5.46 | 9.71 | 0.07 |
| PP_mean_ | Original | 0 | 3.39 | 0.12 () | -18.37 | 15.63 | 34 | 0.15 |
|  | Rank ordered | 0 | 3.39 | 0.11 () | -18.42 | 15.85 | 34.27 | 0.15 |
| PP_SD_ | Original | 0 | 6.13 | -1.14 () | -11.3 | 32.4 | 43.7 | 0.28 |
|  | Rank ordered | 0 | 6.14 | -1.16 () | -11.42 | 32.28 | 43.7 | 0.28 |
| PP_diffABS_ | Original | 0 | 2.61 | -0.29 | -5.73 | 12.43 | 18.16 | 0.12 |
|  | Rank ordered | 0 | 2.59 | -0.31 | -5.71 | 12.45 | 18.16 | 0.12 |

*Descriptive statistics, model residuals for original and rank-ordered data. SD = standard deviation, Med. = median Min = minimum, Max = maximum, SE = standard error*

**Table B.** Shapiro-Wilk (S-W) and Kolmogorov-Smirnov (K-S) normality statistics for original and rank-ordered data across outcome variables.

|  |  | **Shapiro-Wilk** | | **Kolmogorov-Smirnov^†^** | |
| --- | --- | --- | --- | --- | --- |
| **Outcome variable** | **Model data** | **W** | **p-value** | **D** | **p-value** |
| Points | Original | 0.98 | 1.33e-05 | 0.05 | 6.74e-03 |
|  | Rank ordered | 0.98 | 1.55e-05 | 0.04 | 0.03 |
| PP_mean_ | Original | 0.98 | 1.47e-05 | 0.03 | 0.21 |
|  | Rank ordered | 0.98 | 1.12e-05 | 0.03 | 0.26 |
| PP_SD_ | Original | 0.76 | <2.2e-06 | 0.19 | <2.2e-06 |
|  | Rank ordered | 0.76 | <2.2e-06 | 0.19 | <2.2e-06 |
| PP_diffABS_ | Original | 0.97 | 1.76e-08 | 0.06 | 1.45e-04 |
|  | Rank ordered | 0.97 | 1.6e-08 | 0.06 | 6.61e-04 |

*In the S-W normality test, p<0.05 indicates significant evidence to reject the null hypothesis where the data is assumed to be normally distributed. In the K-S normality test, p < 0.05 indicates significant evidence to reject null hypothesis that the data follows a normal distribution. ^†^ = typically applied to n ≥ 50.*

**Table C.** Levene’s test results of all original and rank-ordered data used in model fits*.*

| **Outcome Variable** | **Model data** | **SS** | **MS** | **F value** | **Pr (>F)** |
| --- | --- | --- | --- | --- | --- |
| Points | Original | 1640.8 | 42.07 | 2.54 | 2.56e-06 *** |
|  | Rank-order | 1657.9 | 42.51 | 2.5713 | 1.914e-06 *** |
| PP_mean_ | Original | 29768 | 763.29 | 1.49 | 0.03 * |
|  | Rank-order | 30037 | 770.17 | 1.48 | 0.04 * |
| PP_SD_ | Original | 1053693 | 27018 | 2.08 | 2.5e-04 *** |
|  | Rank-order | 1051004 | 26949 | 2.09 | 2.19e-04 **** |
| PP_diffABS_ | Original | 12009 | 307.93 | 2.15 | 1.23e-04 *** |
|  | Rank-order | 11543 | 295.97 | 2.07 | 2.71e-04 *** |

**Table D.** Skewness and kurtosis results of model residuals. [CI] = 95% confidence interval.

| **Outcome** | **Model data** | **Skewness [CI]** | **Kurtosis [CI]** |
| --- | --- | --- | --- |
| Points | Original | 0.49 [0.22 0.76] | 0.92 [0.43 1.50] |
|  | Rank-order | 0.48 [0.21 0.74] | 0.92 [0.47 1.57] |
| PP_mean_ | Original | -0.27 [-1.05 0.25] | 2.06 [-0.05 5.79] |
|  | Rank-order | -0.26 [-1.04 0.27] | 2.14 [-0.1 5.22] |
| PP_SD_ | Original | 2.51 [2.2 2.86] | 8.12 [5.81 11.51] |
|  | Rank-order | 2.49 [2.19 2.87] | 8.02 [5.92 11.55] |
| PP_diffABS_ | Original | 0.77 [0.49 1.24] | 1.41 [0.32 3.54] |
|  | Rank-order | 0.78 [0.48 1.25] | 1.48 [0.41 3.82] |

**Chi square test model comparisons performed in R**

Points

> anova(mod4c_ME,mod4cr_ME)

Data: data_c

Models:

mod4c_ME: Points ~ Condition + Gender_c + Trial + Condition * PSB_c + Condition * MSOS_c + Condition * CAPSS_c + (1 | Subject)

mod4cr_ME: Points ~ Condition + Gender_c + Trial + Condition * rPSB_c + Condition * rMSOS_c + Condition * rCAPSS_c + (1 | Subject)

npar AIC BIC logLik deviance Chisq Df Pr(>Chisq)

mod4c_ME 16 1940.8 2007.6 -954.41 1908.8

mod4cr_ME 16 1942.7 2009.5 -955.35 1910.7 0 0

PP mean

Models:

mod4c_ME: CP_mean ~ Condition + Gender_c + Trial + Condition * PSB_c + Condition * MSOS_c + Condition * CAPSS_c + (1 | Subject)

mod4cr_ME: CP_mean ~ Condition + Gender_c + Trial + Condition * rPSB_c + Condition * rMSOS_c + Condition * rCAPSS_c + (1 | Subject)

npar AIC BIC logLik deviance Chisq Df Pr(>Chisq)

mod4c_ME 16 2650.2 2716.9 -1309.1 2618.2

mod4cr_ME 16 2649.6 2716.4 -1308.8 2617.6 0.5144 0

PP_SD_

Data: data_c

Models:

mod4c_ME: CP_sd ~ Condition + Gender_c + Trial + Condition * PSB_c + Condition * MSOS_c + Condition * CAPSS_c + (1 | Subject)

mod4cr_ME: CP_sd ~ Condition + Gender_c + Trial + Condition * rPSB_c + Condition * rMSOS_c + Condition * rCAPSS_c + (1 | Subject)

npar AIC BIC logLik deviance Chisq Df Pr(>Chisq)

mod4c_ME 16 3212.8 3279.6 -1590.4 3180.8

mod4cr_ME 16 3210.9 3277.7 -1589.5 3178.9 1.8514 0

Data: data_c

Models:

mod4c_ME: CPDiffCenAbs ~ Condition + Gender_c + Trial + Condition * PSB_c + Condition * MSOS_c + Condition * CAPSS_c + (1 | Subject)

mod4cr_ME: CPDiffCenAbs ~ Condition + Gender_c + Trial + Condition * rPSB_c + Condition * rMSOS_c + Condition * rCAPSS_c + (1 | Subject)

npar AIC BIC logLik deviance Chisq Df Pr(>Chisq)

mod4c_ME 16 2402.2 2469 -1185.1 2370.2

mod4cr_ME 16 2396.2 2463 -1182.1 2364.2 6.0775 0

**Figure A.** Screen of a priori power analysis performed for study to compute sample size in G*Power


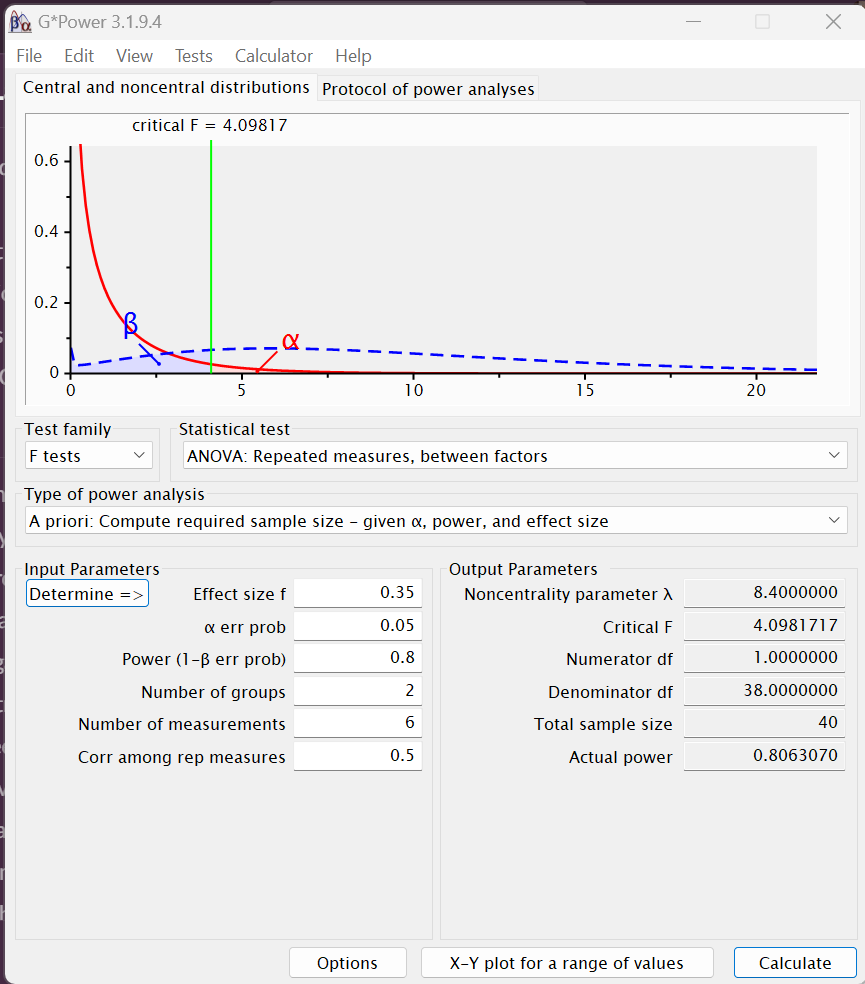


*Graphical user interface of the G*Power application, an open-source tool for statistical power analysis (*[*https://www.psychologie.hhu.de/arbeitsgruppen/allgemeine-psychologie-und-arbeitspsychologie/gpower*](https://www.psychologie.hhu.de/arbeitsgruppen/allgemeine-psychologie-und-arbeitspsychologie/gpower)*).*

**Power analysis of original mixed effects model, post hoc**

*Effect sizes calculated using function ‘F_to_eta2’ in library “effectsize"* [1]*. One-sided 95% confidence intervals (CIs) included. According to Cohen’s recommendations for partial eta squared interpretation as effect sizes,**η^2^ = 0.01 is a small effect size, η^2^ = 0.06 is a medium effect size, and η^2^ = 0.14 is a large effect size* [2]*.*

**Table E.** *Partial eta squared values for the effect size of each predictor and predictor interaction on the outcome variable Points scored.*

| **Variable** | **Partial eta-squared (η^2^)** | **95% CI** |
| --- | --- | --- |
| Condition | 0.04 | [0.02, 1] |
| Trial | 0.04 | [0.01, 1] |
| Gender | 0.36 | [0.16, 1] |
| PSB | 2.02e-03 | [0, 1] |
| MSOS | 0.06 | [0, 1] |
| CAPSS | 1.41e-03 | [0, 1] |
| Condition:PSB | 7.62e-03 | [0, 1] |
| Condition:MSOS | 1.30e-03 | [0, 1] |
| Condition:CAPSS | 0.02 | [0, 1] |

**Table F.** Partial eta squared values for the effect size of each predictor and predictor interaction on the mean paddle portion of ball contacts during game play (PP_mean_).

| **Variable** | **Partial eta-squared (η^2^)** | **95% CI** |
| --- | --- | --- |
| Condition | 0.06 | [0.03, 1] |
| Trial | 0.01 | [0, 1] |
| Gender | 7.12e-04 | [0, 1] |
| PSB | 9.94e-04 | [0, 1] |
| MSOS | 0.03 | [0, 1] |
| CAPSS | 1.68e-03 | [0, 1] |
| Condition:PSB | 6.43e-03 | [0, 1] |
| Condition:MSOS | 6.75e-04 | [0, 1] |
| Condition:CAPSS | 1.41e-03 | [0, 1] |

**Table G.** Partial eta squared values for the effect size of each predictor and predictor interaction on the standard deviation of the paddle portion contacted by the ball (PP_SD_).

| **Variable** | **Partial eta-squared (η^2^)** | **95% CI** |
| --- | --- | --- |
| Condition | 0.07 | [0.04, 1] |
| Trial | 0.01 | [0, 1] |
| Gender | 0.02 | [0, 1] |
| PSB | 0.06 | [0, 1] |
| MSOS | 0.05 | [0, 1] |
| CAPSS | 0.05 | [0, 1] |
| Condition:PSB | 5.01e-03 | [0, 1] |
| Condition:MSOS | 5.84e-04 | [0, 1] |
| Condition:CAPSS | 4.00e-03 | [0, 1] |

**Table H**. Partial eta squared values for the effect size of each predictor and predictor interaction on the outcome variable absolute difference in paddle portion from paddle center (PP_diffABS_).

| **Variable** | **Partial eta-squared (η^2^)** | **95% CI** |
| --- | --- | --- |
| Condition | 0.28 | [0.23, 1] |
| Trial | 0.02 | [0, 1] |
| Gender | 0.08 | [0, 1] |
| PSB | 0.06 | [0, 1] |
| MSOS | 0.02 | [0, 1] |
| CAPSS | 0.04 | [0, 1] |
| Condition:PSB | 1.20e-04 | [0, 1] |
| Condition:MSOS | 0.01 | [0, 1] |
| Condition:CAPSS | 0.01 | [0, 1] |

References

[1] M. S. Ben-Shachar, D. Lüdecke, and D. Makowski, “effectsize: Estimation of Effect Size Indices and Standardized Parameters,” *J. Open Source Softw.*, vol. 5, no. 56, p. 2815, Dec. 2020, doi: 10.21105/joss.02815.

[2] J. Cohen, *Statistical power analysis for the behavioral sciences*, 2. ed., Reprint. New York, NY: Psychology Press, 2009.
